# Abscisic acid enhances DNA damage response through the nuclear shuttling of clathrin light chain 2 in plant cells

**DOI:** 10.1126/sciadv.adt2842

**Published:** 2025-06-13

**Authors:** Jieming Jiang, Qiuhua Zhu, Yang Li, Jiayuan Wu, Chenghuan Zou, Hongbo Li, Xiaohui Lei, Fangyuan Zhang, Yujie Lin, Rui Cao, Ying Cao, Jiawang Mo, Jianbin Lai, Jiangzhe Zhao, Chao Wang, Chengwei Yang

**Affiliations:** ^1^ Guangdong Provincial Key Laboratory of Biotechnology for Plant Development, School of Life Science, South China Normal University, Guangzhou 510631, China.; ^2^ College of Life Sciences, Shaoxing University, Shaoxing 312000, China.; ^3^ College of Life Sciences, Zhejiang Normal University, Jinhua 321004, China.

## Abstract

DNA damage arises from various environmental stresses, and ABA is well known for its roles in plant stress resistance. However, its function in plant DNA damage tolerance remains unclear. In this study, we showed that ABA supplementation significantly enhances plant tolerance to DNA-damaging treatments. SnRK2.2 and SnRK2.3 kinases in the ABA signaling pathway are pivotal in this process. These kinases interact with clathrin light chain 2 (CLC2), facilitating its phosphorylation and nuclear translocation in response to Zeocin and ABA treatment. In the nucleus, CLC2 interacts with ADA2b, an adaptor protein crucial for recruiting SMC5/6 complex to the double-strand break (DSB) sites. The enhanced nuclear localization of CLC2 is essential for the accurate localization of ADA2b at DSB sites. Collectively, our study uncovers that ABA enhances plant DNA damage tolerance with a distinct function of CLC2 in genomic stability maintaining, thereby improving our understanding of DNA damage tolerance mechanisms in plants.

## Teaser

ABA signaling is required for full activation of DNA damage response.

## INTRODUCTION

All living organisms are constantly affected by endogenous or exogenous factors that result in various DNA lesions (*1*). DNA double-strand breaks (DSBs), induced by exogenous agents such as Zeocin, methyl methanesulfonate (MMS), and ionizing radiation or by endogenous reactive oxygen species (ROS), are among the most cytotoxic forms of DNA damage. They disrupt genomic stability, reduce cell viability, and can even lead to cancer in animals (*2*, *3*). Eukaryotic cells respond to DNA damage through cell cycle control, transcriptional reprogramming, DNA repair, and programmed cell death (PCD) when they fail to repair the damage (*4*). DNA repair sequentially recruits repair factors to DSB sites to maintain genome integrity. Eukaryotic cells use two evolutionarily conserved pathways, nonhomologous end joining (NHEJ) and homologous recombination (HR), for DSB repair (*5*). NHEJ is a more error-prone process initiated by the DNA-dependent protein kinase (DNA-PK) holoenzyme, which directly joins the ends of DSBs. In contrast, HR is a highly accurate repair mechanism that uses homologous sequences as templates, functioning predominantly during the S and G_2_ phases of the cell cycle to ensure genomic stability (*6*). During this process, efficient and precise repair relies on the orchestrated recruitment of DNA repair proteins. For example, the structural maintenance of chromosome 5/6 (SMC5/6) complex is essential for the repair of DSBs through the HR pathway (*7*, *8*). DNA damage enhances the expression levels of SWI3B, which helps dissociate the SMC5/6 complex from chromosomes (*9*). ADA2b, a conserved component in the Spt-Ada-Gcn5 acetyltransferase complex, interacts with SMC5 by competing with SWI3B and subsequently recruits the complex to DSB sites under the guidance of the diRNA/AGO2-IDN2-CDC5 protein scaffold (*9*–*12*). A recent study demonstrated that *Arabidopsis* cryptochromes directly interact with ADA2b and SMC5, thereby promoting DSB repair in a blue light-dependent manner (*13*). Together, these proteins collaborate to maintain the genome integrity, safeguarding cellular functionality and the overall organismal health (*14*).

Plant hormones are crucial for DNA repair under stressful conditions (*15*, *16*). Abscisic acid (ABA) is a pivotal hormone that accumulates under stress conditions to enhance plant tolerance (*17*). Previous studies have also showed that stress conditions lead to the up-regulation of ROS, which, in turn, activate the ABA signaling pathway (*18*–*20*). ROS contributes to a positive feedback loop, where ABA further promotes ROS production, thereby enhancing the overall stress response (*21*–*24*). The ABA signaling pathway is regulated by the interaction between protein phosphatase of group C (PP2Cs) and subclass III SNF1-related protein kinase 2 (SnRK2s). In the absence of ABA, PP2Cs interact with SnRK2 to inhibit ABA signaling. In the presence of ABA, it binds the receptors pyrabactin resistance 1/PYR1-like/regulatory components of ABA receptors (PYR/PYL/RCARs), causing structural changes that allow them to interact with PP2Cs, thereby restoring the activity of SnRK2s (*25*). Among the signaling pathway, the double *abi1-2/abi2-2* mutant showed higher sensitivity to ABA treatment and enhanced resistance to drought stress (*26*). In contrast, double mutant of *SnRK2.2* and *SnRK2.3* exhibits a strong ABA-insensitive phenotype in seed germination and root growth (*27*), while the *snrk2.2/2.3/2.6* triple mutants almost completely impair ABA responses in plants (*28*–*30*). In addition to its role in abiotic stress responses, ABA signaling has also been implicated in coordinating cellular mechanisms under genotoxic stress conditions (*31*). Subsequent studies revealed that DNA replication factor C1 plays a critical role in ABA-mediated HR repair in *Arabidopsis* (*32*). Moreover, mutation of the catalytic subunit of DNA polymerase ε, POL2a, results in an ABA overly sensitive phenotype (*33*), and some HR repair–defective mutants show enhanced sensitivity to ABA (*34*). A recent study further demonstrated that histone variant H2A.X functions in the DNA damage–coupled ABA signaling pathway (*35*). Collectively, these studies strongly suggest the relationship between ABA and DNA damage tolerance. However, the underlying mechanisms of ABA signaling in this process require further investigation.

Clathrin is well known for its roles in clathrin-mediated endocytosis (CME), a vesicular transport process that is crucial for signal transduction, nutrient import, and synaptic vesicle trafficking (*36*). Clathrin lattice, triskelions comprising three heavy chains (CHCs) and three light chains (CLCs), facilitates vesicle budding and formation (*37*, *38*). Clathrin-coated vesicles regulate cargo trafficking between the plasma membrane, endosomes, and the Golgi apparatus (*39*), enabling various cellular processes. In plants, the loss of *CLC2* and *CLC3* disrupts auxin-regulated endocytosis of pin-formed (PIN) proteins, leading to pleiotropic developmental defects, including reduced male fertility (*40*). The double mutant of *CLC2* and *CLC3* also shows accelerated cell death due to elevated levels of ROS and salicylic acid (SA) (*41*). Furthermore, CLC2 interacts with autophagy-related gene 8 (ATG8) and localizes to the cisternal membrane, facilitating Golgi reassembly after recovery from heat stress (*42*). In animals, clathrin plays additional roles beyond trafficking, including mitotic spindle organization during cell division and promoting cancer cell proliferation through CHC phosphorylation (*43*, *44*). Inhibition of endocytosis has been linked to DNA damage responses that promote apoptosis (*45*), while DNA damage itself disrupts endocytosis via poly(ADP-ribose) polymerase 1–mediated poly(ADP-ribose) production, which inhibits Rab5 activity, impairs nutrient uptake, and contributes to cell death (*46*). Moreover, nucleus localization of clathrin also enables interaction with the tumor suppressor p53, enhancing p53-dependent transcription (*47*, *48*). Collectively, these findings highlight the multifaceted roles of clathrin in cellular function and DNA stress responses, extending far beyond its canonical role in vesicular.

In this study, we found that ABA supplementation significantly enhances plant resistance to DNA damage through its signaling pathway. Further analysis revealed that SnRK2.2 and SnRK2.3 interact with CLC2 and promote its nuclear shuttling through phosphorylation in response to DNA damage treatment. CLC2 then interacts with the DNA damage response factor ADA2b in the nucleus, facilitating in its precise localization at DSB sites. This study elucidates the mechanism of ABA-related DNA damage response and highlighted the role of clathrin in maintaining genomic stability, deepening our understanding of DNA damage response mechanisms.

## RESULTS

### ABA enhances plant resistance to DNA damage

**Fig. 1.**
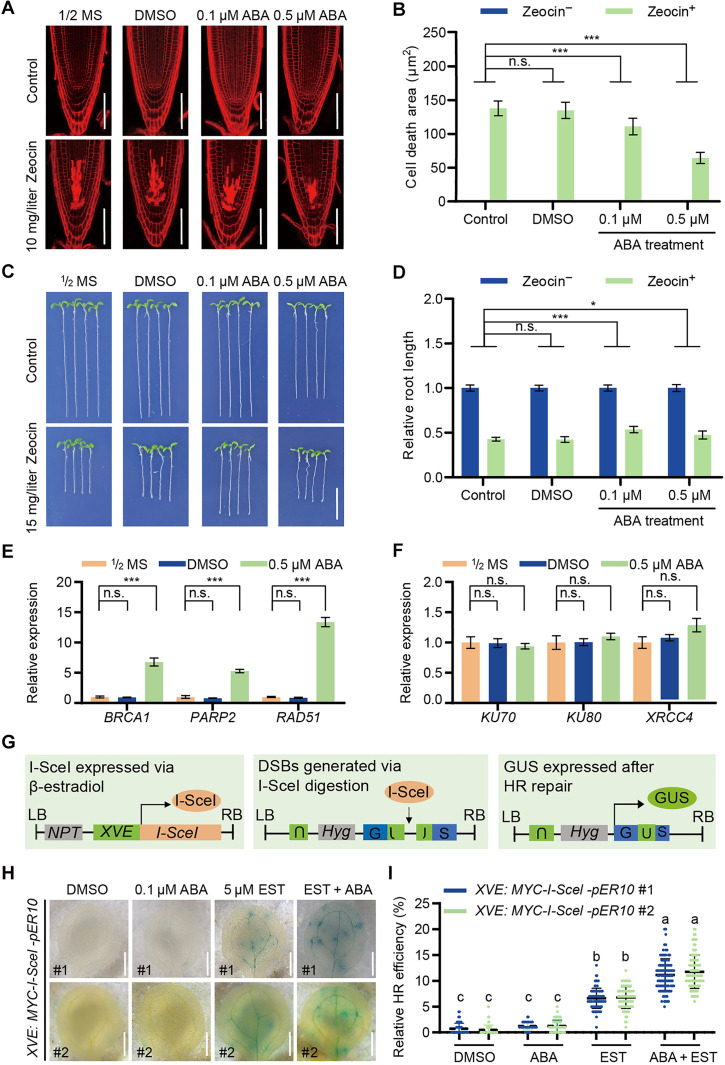
ABA enhances plant resistance to DNA damage. (**A** and **B**) Effects of ABA on root meristems upon DNA damage in wild-type seedlings. Five-day-old seedlings were transferred to ABA treatment for 8 hours before further treated with or without indicated ABA and Zeocin for 12 hours. Representative images from three biologically independent experiments are shown in (A) by PI staining. Scale bars, 50 μm. The quantitative data shown in (B) are means ± SD. Significant differences are indicated using two-way analysis of variance (ANOVA) analysis (Dunnett’s multiple comparisons test, *n* = 15). ***P < 0.001; n.s., no significance. (**C** and **D**) Effects of ABA on primary root growth upon DNA damage in wild-type seedlings. Five-day-old seedlings were transferred to ABA treatment for 8 hours before further treated with ABA and Zeocin for another 48 hours. Representative images from are shown in (C). The quantitative data shown in (D) are means ± SD. Scale bar, 1 cm. Significant differences were determined using two-way ANOVA test (Dunnett’s multiple comparisons test, *n* = 15). ***P < 0.001 and *P < 0.05. (**E** and **F**) Transcriptional analysis of DNA repair genes via quantitative reverse transcription polymerase chain reaction in 7-day-old seedlings treated with ABA for 12 hours. The presented data are means ± SD from three technical replicates. Student’s *t* test. ***P < 0.001. (**G**) Schematic representation of HR reporter system. The reporter line contains an I-SceI site within the *GUS* gene and a donor sequence (U). β-Estradiol induces I-SceI expression, creating a DSB. HR repair restores the functional GUS gene. LB, left border; RB, right border. (**H** and **I**) Representative GUS staining images of cotyledons (H) and quantification of relative HR efficiency (I) are shown. Scale bars, 0.5 cm. The number of blue sectors was counted, and the data are presented as means ± SD (*n* = 100). Statistical significance was determined using one-way ANOVA (Tukey’s multiple comparisons test) with different letters indicated above the columns, *P* < 0.001. DMSO, dimethyl sulfoxide.

Genome integrity is crucial for the proper function and survival of eukaryotic cells (*49*). In plants, genome integrity is also regulated by multiple hormones (*15*, *16*). Although previous studies have revealed a relationship between ABA and genotoxic stress (*31*), the effect of ABA on the DNA damage response remains unclear. Therefore, we examined the cell death areas in the root meristems using propidium iodide (PI) staining because PCD often occurs when damaged DNA cannot be repaired. The result showed that ABA significantly suppressed the cell death induced by both MMS and Zeocin at both concentrations tested (Fig. 1, A and B, and fig. S1, A and B). Moreover, higher ABA concentration exhibited a more pronounced inhibitory effect on cell death, suggesting that ABA contributes to enhanced DNA damage tolerance.

To further validate this observation, we assessed root growth under the treatment with both agents in the presence of ABA. The results revealed that 0.1 μM ABA, which had no adverse effect on root growth, significantly promoted the root elongation under Zeocin and MMS treatment (Fig. 1, C and D, and fig. S1, C and D). However, as the ABA concentration increased to 0.5 μM, this beneficial effect was suppressed, as higher ABA levels inhibited the root growth even under the control conditions. Despite its inhibitory effect on overall root growth, ABA still exhibited a protective role by alleviating damaging agent-induced growth suppression, highlighting its dual regulatory function in coordinating growth and stress responses.

ABA was previously reported to influence the expression of HR genes (*33*, *34*, *50*); therefore, the inhibition of cell death area by ABA in the root meristems might result from change in DNA damage response. To test this hypothesis, we analyzed the expression of both HR- and NHEJ-related genes under ABA treatment. The results showed that ABA treatment significantly up-regulated HR-related gene expression (Fig. 1E) but had no significant effect on NHEJ-related gene expression (Fig. 1F), suggesting that ABA selectively enhances HR-related DDR pathways.

To further confirm whether ABA directly enhances the frequency of HR, we modified and used a reporter system in *Arabidopsis* based on an estradiol-inducible expression of I-SceI endonuclease, which creates DSBs at a specific site and recovers the *GUS* expression after HR repair (Fig. 1G) (*51*). Using this system, we observed a pronounced increase in HR repair efficiency only when both ABA and estradiol were applied simultaneously (Fig. 1, H and I). These findings provide strong evidence that ABA enhances plant resistance to DNA damage by promoting the activation of DDR through the HR repair system, further supporting its pivotal role in maintaining genome integrity under stress conditions.

To further explore the relationship between ABA and DNA repair, we assessed the ABA content and ROS levels under DNA damage conditions induced by Zeocin and MMS. Our results revealed that DNA damage treatment significantly increased free ABA (fig. S2A) and ROS levels (fig. S2B) in plants. In addition, the ROS accumulation induced by MMS and Zeocin was accompanied by a corresponding increase expression of marker genes downstream of the ABA signaling pathway (fig. S2C). These findings suggest that MMS and Zeocin treatment can activate both ROS and ABA signaling pathways, providing a mechanistic basis for further investigation into how ROS and ABA signaling converge to mediate the cellular response to genotoxic stress.

### ABA signaling pathway is involved in Zeocin-induced response

**Fig. 2.**
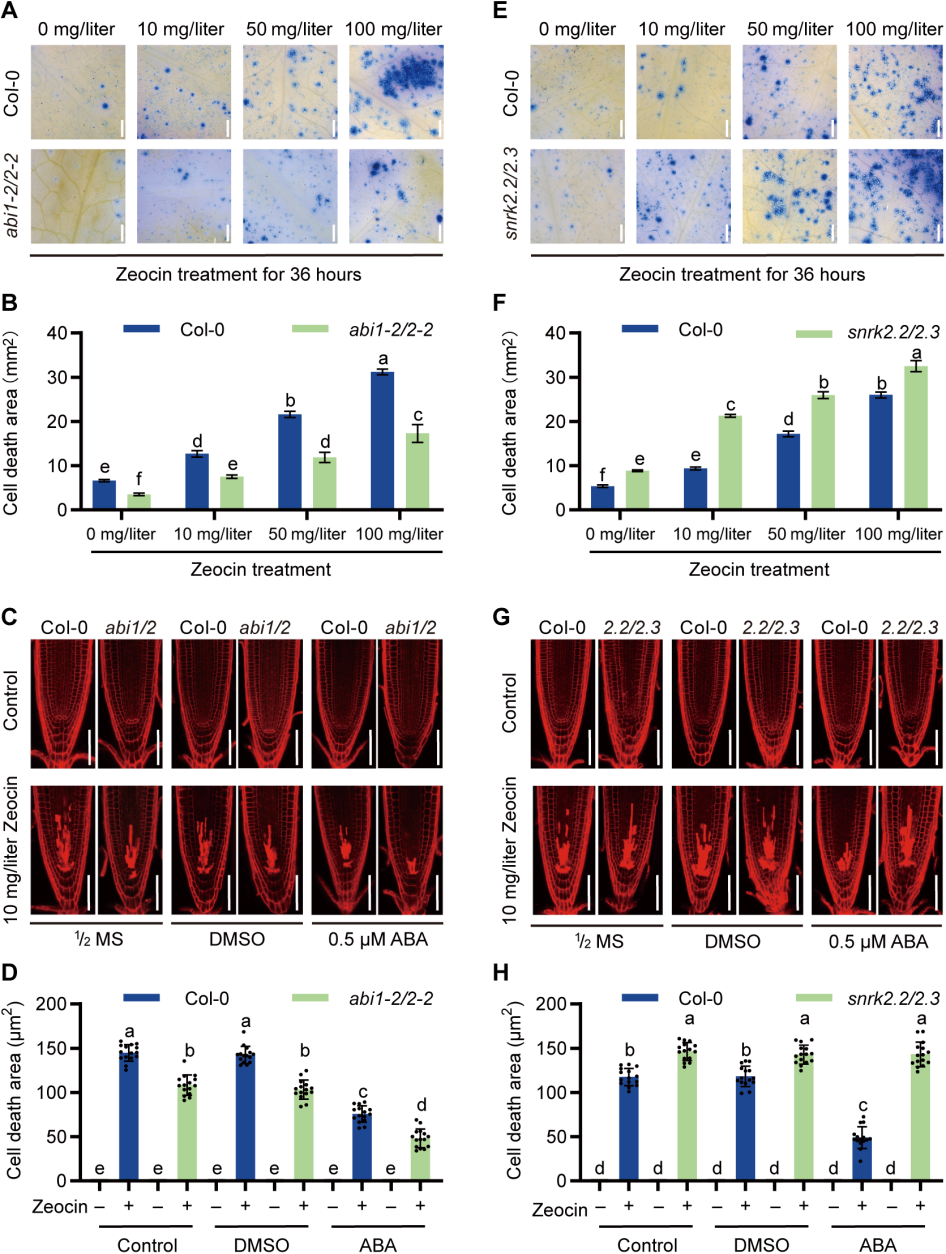
ABA signaling pathway is involved in Zeocin-induced response. (**A** and **B**) Detection of Zeocin sensitivity in *abi1-2/abi2-2* mutant plants. Four-week-old leaves were transferred to ^1^/_2_ MS medium with or without Zeocin for 36 hours and then subjected to trypan blue staining. Images shown in (A) are representative results from three biologically independent experiments. Scale bars, 0.5 mm. The cell death areas are shown in (B). The data are means ± SD from three leaves with significant differences between Columbia-0 (Col-0) and mutant seedlings determined using one-way ANOVA test (Tukey’s multiple comparisons test). *P* < 0.001. (**C** and **D**) Detection of cell death in the *abi1-2/abi2-2* mutant in ABA-mediated DNA damage tolerance. (C) The 5-day-old seedlings were transferred to medium with or without ABA for 8 hours and then incubated in medium with or without ABA and Zeocin for 12 hours. Scale bars, 50 μm. (D) Cell death areas were analyzed. Statistical data with significant differences (means ± SD; *n* = 15) are shown using one-way ANOVA analysis (Tukey’s multiple comparisons test). Significant differences are indicated with different letters above the columns. *P* < 0.001. (**E** and **F**) Detection of Zeocin sensitivity in *snrk2.2/snrk2.3* mutant plants. Four-week-old leaves were transferred to ^1^/_2_ MS medium containing or lacking Zeocin for 36 hours. Representative results were shown in (E). Scale bars, 0.5 mm. The cell death areas were quantified in (F). The data are presented (means ± SD; *n* = 3) with significant differences determined using a one-way ANOVA (Tukey’s multiple comparisons test). *P* < 0.001. (**G** and **H**) Detection of cell death in the *snrk2.2/snrk2.3* mutant in ABA-mediated DNA damage tolerance. Representative images shown in (G) were from three biologically independent experiments. Scale bars, 50 μm. Statistical data for the cell death areas (means ± SD; *n* = 15) were shown in (H). Significant differences are indicated using one-way ANOVA analysis (Tukey’s multiple comparisons test) and marked with different letters above the columns. *P* < 0.001.

Having shown that ABA enhances plant resistance to DNA damaging agents, it is crucial to determine the role of ABA signaling pathway in this process. We started to test the Zeocin-induced sensitivity from the available *abi1*-*2/abi2*-*2*, double mutant of PP2C family, which was previously reported to be ABA sensitive (*26*). With increasing concentrations of Zeocin, the *abi1-2/abi2-2* double mutant exhibited a more tolerant phenotype compared to the wild type, as evidenced by smaller areas of death cell in leaves stained by trypan blue (Fig. 2, A and B). Consistently, the double mutant showed smaller areas of cell death in the presence of Zeocin compared to the wild type, and these areas were further reduced with ABA supplementation (Fig. 2, C and D), providing evidence that ABI1 and ABI2 act as negative regulators in this process. Moreover, the root length of the double mutant was longer than that of wild type, and the difference became more pronounced after DNA damage treatment. The application of ABA further enhanced root growth in the double mutant following Zeocin treatment (fig. S3, A and B), further supporting the notion that ABI1 and ABI2 are negative regulators in the Zeocin-induced response.

In contrast, the *snrk2.2/2.3* double mutant was more sensitive to Zeocin. Trypan blue staining revealed significantly larger areas of cell death in the leaves of the double mutant compared to the wild type, demonstrating its compromised ability to tolerate DNA damage (Fig. 2, E and F). This suggests that SnRK2.2 and SnRK2.3 act as positive regulators in the Zeocin-induced response. To further investigate, we examined the root meristem regions. Consistent with the findings in leaves, the *snrk2.2/2.3* double mutant displayed markedly higher levels of cell death in the root meristems compared to the wild type after Zeocin treatments (Fig. 2, G and H). Notably, the application of ABA failed to mitigate cell death in the mutant root meristems (Fig. 2, G and H), suggesting that the absence of SnRK2.2 and SnRK2.3 impairs ABA-mediated DNA damage tolerance in the root. In addition, root growth analysis revealed that the *snrk2.2/2.3* double mutant was more severely affected by Zeocin treatment than the wild type, with the root length differences becoming more pronounced (fig. S3, C and D). ABA supplementation could not rescue the root growth defect in the double mutant (fig. S3, C and D), further indicating that SnRK2.2 and SnRK2.3 are integral to the ABA-mediated DNA damage tolerance.

### SnRK2s interacts with CLC2 for Zeocin-induced response

**Fig. 3.**
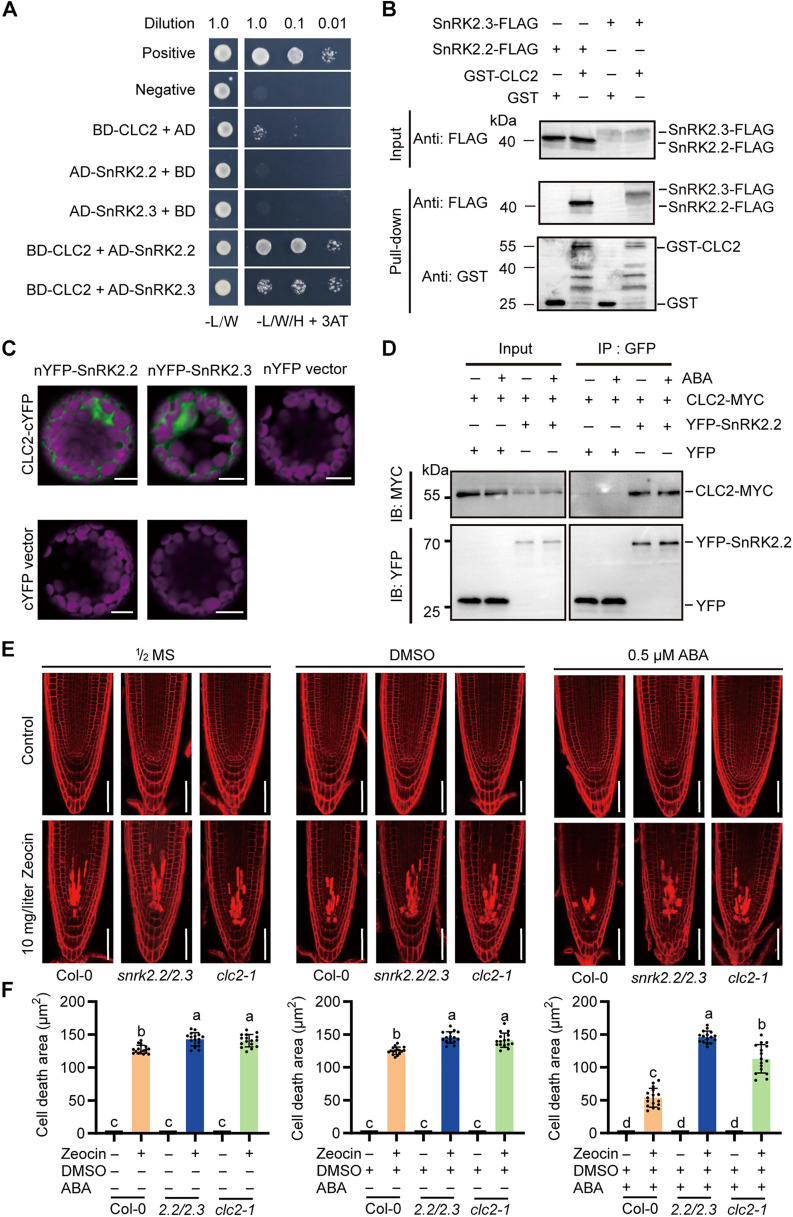
SnRK2s interacts with CLC2 Zeocin-induced response. (**A**) The interaction between SnRK2s and CLC2 was detected using yeast two-hybrid assay. SnRK2s were fused with activation domain (AD), and CLC2 was fused with binding domain (BD). The interaction was determined on SD-L-W-H medium containing 30 mM 3-amino-1,2,4-triazole (3-AT). (**B**) In vitro pull-down assay was used to detected the interaction of SnRK2s and CLC2. SnRK2s were fused with FLAG tag, while CLC2 was fused with GST tag. SnRK2s precipitated with GST-CLC2 and free GST (negative control) were detected using an anti-FLAG antibody. (**C**) Interaction of nYFP-SnRK2s and CLC2-cYFP in protoplasts via BiFC assay. Vectors were cotransformed into wild-type protoplasts obtained from 3-week-old seedlings and incubated for 24 hours before recording in a confocal microscope. Scale bars, 10 μm. (**D**) The association of SnRK2s and CLC2 was measured using coimmunoprecipitation assay in plant cells. CLC2-MYC was coexpressed with YFP-tagged SnRK2s or free YFP (negative control) in protoplasts. Immunoprecipitations (IP) were conducted using anti-GFP agarose and total protein. The input and IP protein signals were obtained using anti-GFP and anti-MYC antibodies, respectively. IB, immunoblotting. (**E** and **F**) Detection of ABA-mediated DNA repair in *clc2-1* mutant plants. (E) Before transferred to medium with or without 0.5 μM ABA for 8 hours, the indicated seedlings were vertically grown in ^1^/_2_ MS for 5 days. Then, the seedlings were further transferred to medium with or without 0.5 μM ABA and Zeocin (10 mg/liter) for 12 hours. The experiment used DMSO as negative controls. PI staining was used to observe the cell death areas in the root meristems. Scale bars, 50 μm. (F) Cell death areas were quantified by ImageJ software (means ± SD; *n* = 15). One-way ANOVA analysis was used to estimate the significant differences between the control and treatments (Tukey’s multiple comparisons test). Significant differences are indicated by the different letters marked above the columns. *P* < 0.001.

Previous reports have showed that endocytic proteins can associate with nuclear factors, changing their localization and activity and suggesting a novel role for clathrin in the nucleus (*52*). Recent studies have demonstrated that clathrin heavy chains are present in the nucleus and participate in transcriptional coactivation through interaction with the tumor suppressor p53, suggesting a potential role in DNA damage response (*37*–*40*). Having shown that SnRK2.2 and SnRK2.3 were important for ABA-mediated damage tolerance, we next screened their interactors using a small library containing plant CLC1, CLC2, CLC3, and CHC1. We found that only CLC2 physically interacted with SnRK2.2 and SnRK2.3 in yeast cells (Fig. 3A). The interaction was further confirmed by a pull-down assay that CLC2 was tagged with glutathione *S*-transferase (GST) and precipitated specifically with the SnRK2.2-FLAG and SnRK2.3-FLAG in vitro (Fig. 3B). In addition, a further bimolecular fluorescence complementation (BiFC) experiment in protoplasts demonstrated that nYFP-SnRK2s and CLC2-cYFP reconstituted yellow fluorescent protein (YFP) fluorescence in both the cytoplasm and nuclei, indicating their potential interaction in vivo (Fig. 3C). To validate this interaction in plants, a coimmunoprecipitation assay was performed, which confirmed that both SnRK2.2 and SnRK2.3 bind to CLC2 in plant cells. However, the presence of ABA did not enhance the strength of this interaction (Fig. 3D and fig. S4).

Given that CLC2 colocalized with SnRK2.2 and SnRK2.3 in both the cytoplasm and nuclei, we hypothesized that CLC2 might be involved in the ABA-mediated DNA damage tolerance. To verify this assumption, the DNA damage sensitivity in *clc2*-*1* mutant was examined using *snrk2.2/2.3* double mutant as a control. As was shown, trypan blue staining revealed that the leaves of the *clc2-1* mutant were significantly more sensitive to Zeocin compared to the wild type (fig. S5, A and B). While ABA supplementation did not enhance cell death in either mutant, it alleviated cell death in the *clc2-1* mutant. However, the difference in sensitivity between *clc2-1* and the wild type remained statistically significant (fig. S5, A and B). Similarly, root meristem assays showed that cells in the root of the *clc2-1* mutant were more sensitive to Zeocin treatment. Even in the presence of ABA, the reduction in cell death in the root meristems of *clc2-1* did not reach wild-type levels (Fig. 3, E and F), indicating a role for CLC2 in ABA-mediated Zeocin tolerance. To further validate the involvement of ABA and CLC2 in DNA damage tolerance, we performed a root growth assay. The roots of *clc2-1* seedlings were significantly more sensitive to Zeocin treatment compared to the wild type (fig. S6, A and B). Consistently, the impaired root growth in *clc2-1* was partially recovered upon ABA supplementation, although not to the extent observed in wild-type seedlings (fig. S6, A and B). To further examine their genetic relationship, root length measurements among *snrk2.2/2.3/clc2-1* triple mutant, **snrk2.2/2.3*,* and *clc2-1* showed that the root length of triple mutant was indistinguishable from that of *snrk2.2/2.3*, suggesting that SnRK2.2/2.3 may act upstream of CLC2 (fig. S7, A and B). Together, the enhanced sensitivity of *clc2-1* mutant to DNA damage, along with its partial recovery upon ABA treatment, supports its role in maintaining genomic stability under DNA stress. These findings suggest that CLC2 and SnRK2.2/2.3 are involved in ABA-mediated DNA damage tolerance.

### Phosphorylation facilitates nuclear shuttling of CLC2

Given that CLC2 may participate in DNA damage response, one possibility is that it regulates genomic stability by influencing vesicular transport among endomembrane systems. Alternatively, because of its nuclear localization, CLC2 might directly participate in DNA damage response through interactions with other DNA repair factors. Previous studies have shown that the internalization of FM4-64 (*N*-(3-triethylammoniumpropyl]-4[6-(4-(diethylamino)phenyl) hexatrienyl] pyridinium dibromide), a lipophilic styryl dye, is dependent on CME. To assess whether endocytosis is affected, we used FM4-64 to detect internalization in the indicated cells. The results indicated that neither the *CLC2* mutant nor the *SnRK2.2/2.3* double mutant influenced cell endocytosis in plants under normal conditions (fig. S8., A and B). Although DNA damage significantly suppressed cell endocytosis, no differences were observed among the genotypes (fig. S8, A and B). This suggests that the involvement of SnRK2s and CLC2 in the Zeocin-induced response is independent of endocytosis.

**Fig. 4.**
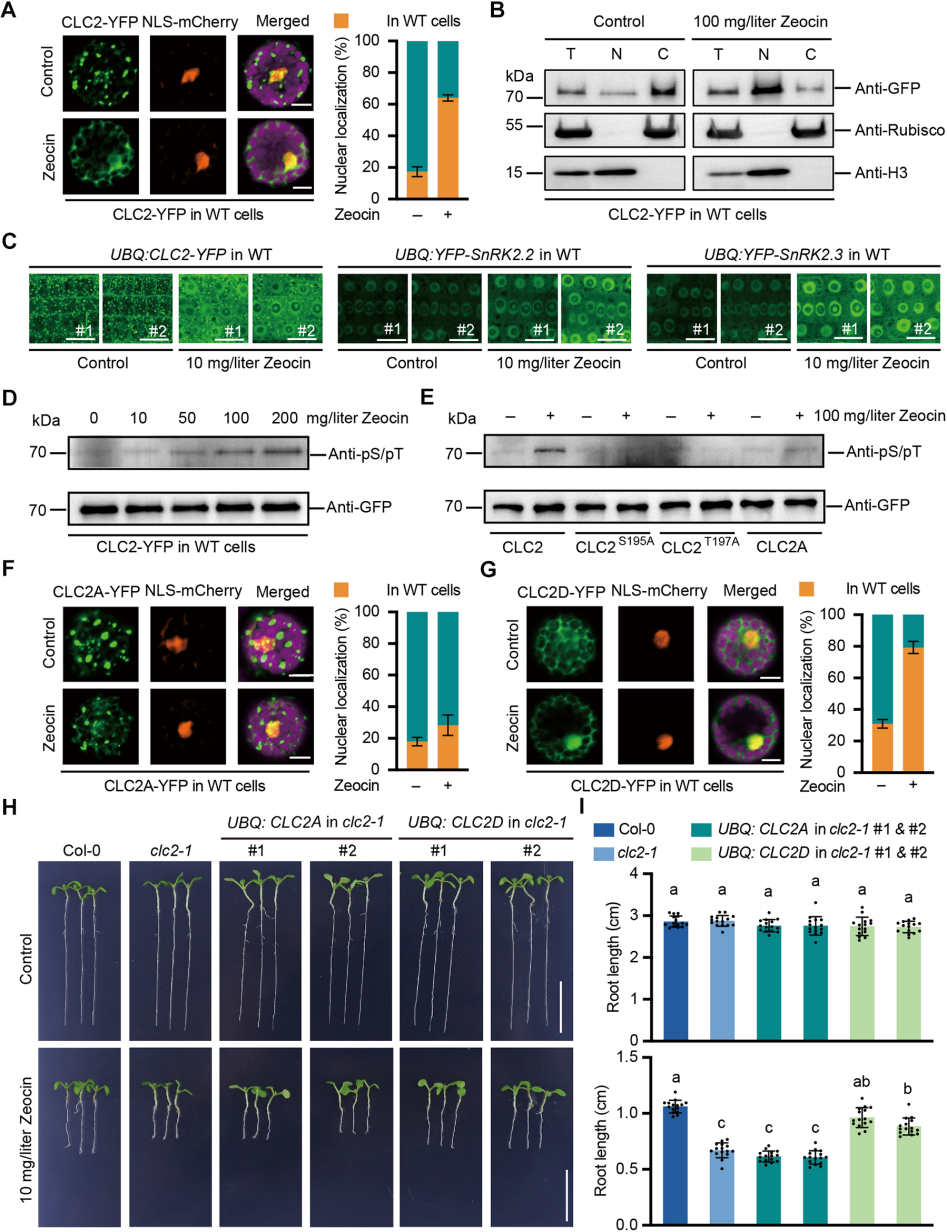
Phosphorylation facilitates nuclear shuttling of CLC2. (**A**) Localization of CLC2-YFP in wild-type (WT) cells with and without DNA damage. Representative images were shown. Scale bars, 10 μm. Percentages (means ± SD, *n* = 100) of cells with and without nucleus signals are from three independent experiments. (**B**) The distribution of CLC2-YFP is determined. Protoplasts expressing CLC2-YFP were incubated with or without Zeocin for 24 hours. The CLC2-YFP was detected by an anti-GFP antibody. Anti-Rubisco and anti-H3 antibodies were used for marking the cytoplasmic and nucleus fractions, respectively. T, Total; N, Nucleus; C, Cytoplasm. (**C**) The localization of CLC2-YFP and YFP-SnRK2s in intact roots of transgenic wild-type seedlings. Five-day-old seedlings were treated with or without Zeocin for 12 hours before recorded. Representative results from three independent experiments are shown. Scale bars, 15 μm. (**D**) Phosphorylation levels of CLC2-YFP upon DNA damage. Protoplasts expressing CLC2-YFP were incubated with or without Zeocin for 24 hours before phosphorylation detection. (**E**) Detection of phosphorylation sites of CLC2-YFP upon DNA damage. Protoplasts expressing CLC2-YFP or its mutated forms were incubated with or without Zeocin for 24 hours. The samples were precipitated using anti-GFP agarose from total protein. YFP and phosphorylation signals were obtained using anti-GFP and anti-phosphoserine/threonine antibodies, respectively. (**F** and **G**) Subcellar localization of CLC2A-YFP and CLC2D-YFP in wild-type cells with and without DNA damage. Representative images were shown. Scale bars, 10 μm. Percentages (means ± SD, *n* = 100) of cells with and without nucleus signals are from three independent experiments. (**H** and **I**) Phenotypes of *CLC2A* and *CLC2D* supplementary lines. Seedlings were grown with or without Zeocin for 7 days before recording. Representative images from three biologically independent experiments are shown in (H). Scale bars, 1 cm. Quantitative data of root length in (I) are presented as the means ± SD (*n* = 15). Significant differences are indicated with different letters above the columns. *P* < 0.001, Tukey’s multiple comparisons test.

To further elucidate how CLC2 participates in this process, we fused CLC2 with YFP and expressed it in protoplasts alongside Nuclear Localization Sequence (NLS)-mCherry as a nuclear marker. Under normal conditions, CLC2-YFP was evenly distributed in the trans-Golgi network/early endosome (TGN/EE) (Fig. 4A). However, upon Zeocin treatment, CLC2-YFP no longer localized to the TGN/EE but accumulated in the nuclei of most cells (Fig. 4A). A nuclear fractionation assay further confirmed that DNA damage induces the nuclear localization of CLC2 (Fig. 4B), supporting the notion that CLC2 may directly contribute to the DNA damage response. To validate these findings in intact plants, we transformed wild-type seedlings with CLC2-YFP or SnRK2s-YFP. Two transgenic lines with similar expression levels were selected for analysis (fig. S9). Consistent with our observations in protoplasts, CLC2-YFP colocalized with the membrane system under normal conditions and translocated to the nuclei upon DNA damage treatment (Fig. 4C and fig. S9A), demonstrating that both agents induced similar effects on CLC2 subcellular localization. In contrast, SnRK2.2 and SnRK2.3 were localized in both the nuclei and cytoplasm, with their localization remaining unchanged under the treatment with either Zeocin (Fig. 4C) or MMS (fig. S9, B and C).

SnRK2s have been reported to regulate the nuclear localization of FREE1 through phosphorylation in the ABA signaling pathway (*53*). Given that SnRK2.2 and SnRK2.3 remain immobile during Zeocin-induced response, we hypothesized that the nuclear shuttling of CLC2 may be mediated by its phosphorylation. To test this, we expressed CLC2-YFP in protoplasts and examined its phosphorylation status following Zeocin treatment. The results indicated that CLC2 phosphorylation was notably enhanced (Fig. 4D). While CLC2 was previously identified as being phosphorylated at S195 and T197 (*54*), the specific kinases responsible and the functional significance of these phosphorylation sites remained unclear. To address this, we analyzed the impact of mutating these sites. Mutation of either S195 or T197 to alanine reduced the phosphorylation levels of CLC2, and the double mutant (CLC2A) completely abolished phosphorylation under DNA damage conditions (Fig. 4E). These findings suggest that phosphorylation at S195 and T197 is crucial for the role of CLC2 in Zeocin-induced response.

Given that phosphorylation of CLC2 is up-regulated under Zeocin treatment, the next question is whether the phosphorylation regulates the localization of CLC2. Therefore, both sites were mutated to alanine (CLC2A) or aspartate (CLC2D) and fused with YFP for expression in protoplasts. The results indicated that CLC2A-YFP localized to TGN/EE and maintained this localization during Zeocin treatment, with only few cells showing YFP signals in the nuclei (Fig. 4F). In contrast, CLC2D-YFP lost its TGN/EE localization, and Zeocin treatment significantly enhanced its nuclear shuttling (Fig. 4G). This conclusion was further supported by stable complementary lines selected for similar expression levels (fig. S10). As was shown, CLC2A prevented nuclear localization under the treatment of Zeocin (fig. S10A). In contrast, *CLC2D*-*YFP* transgenic lines exhibited nuclear localization under normal conditions, with DNA damage further enhancing its nuclear shuttling (fig. S10B). In addition, root length analysis showed that neither CLC2A nor CLC2D had an effect on root growth in the *clc2*-*1* mutant; however, the sensitivity of *clc2-1* to Zeocin treatment was not rescued by overexpression of *CLC2A* but was completely inhibited by overexpression of *CLC2D* (Fig. 4, H and I). These findings provide further evidence that phosphorylation of CLC2 at S195 and T197 may be essential for its role in DNA damage response.

### SnRK2s enhances phosphorylation of CLC2

To determine whether the nuclear localization of CLC2 is regulated via the ABA signaling pathway, we further examined the phosphorylation of CLC2 following ABA treatment. The results showed that ABA notably activated CLC2 phosphorylation (fig. S11A), but this effect was abolished when the phosphorylation sites were mutated (fig. S11B). Nuclear localization analysis in similar expression lines indicated that ABA substantially enhanced the nuclear localization of CLC2 (fig. S12A). However, this effect was abolished when CLC2 was expressed in the *snrk2.2/2.3* mutant background (fig. S12B). Compared to CLC2A, the CLC2D restored its nuclear localization in the *snrk2.2/2.3* under ABA treatment (fig. S12, C and D), confirming that SnRK2.2 and SnRK2.3 mediate CLC2 phosphorylation during the ABA response.

**Fig. 5.**
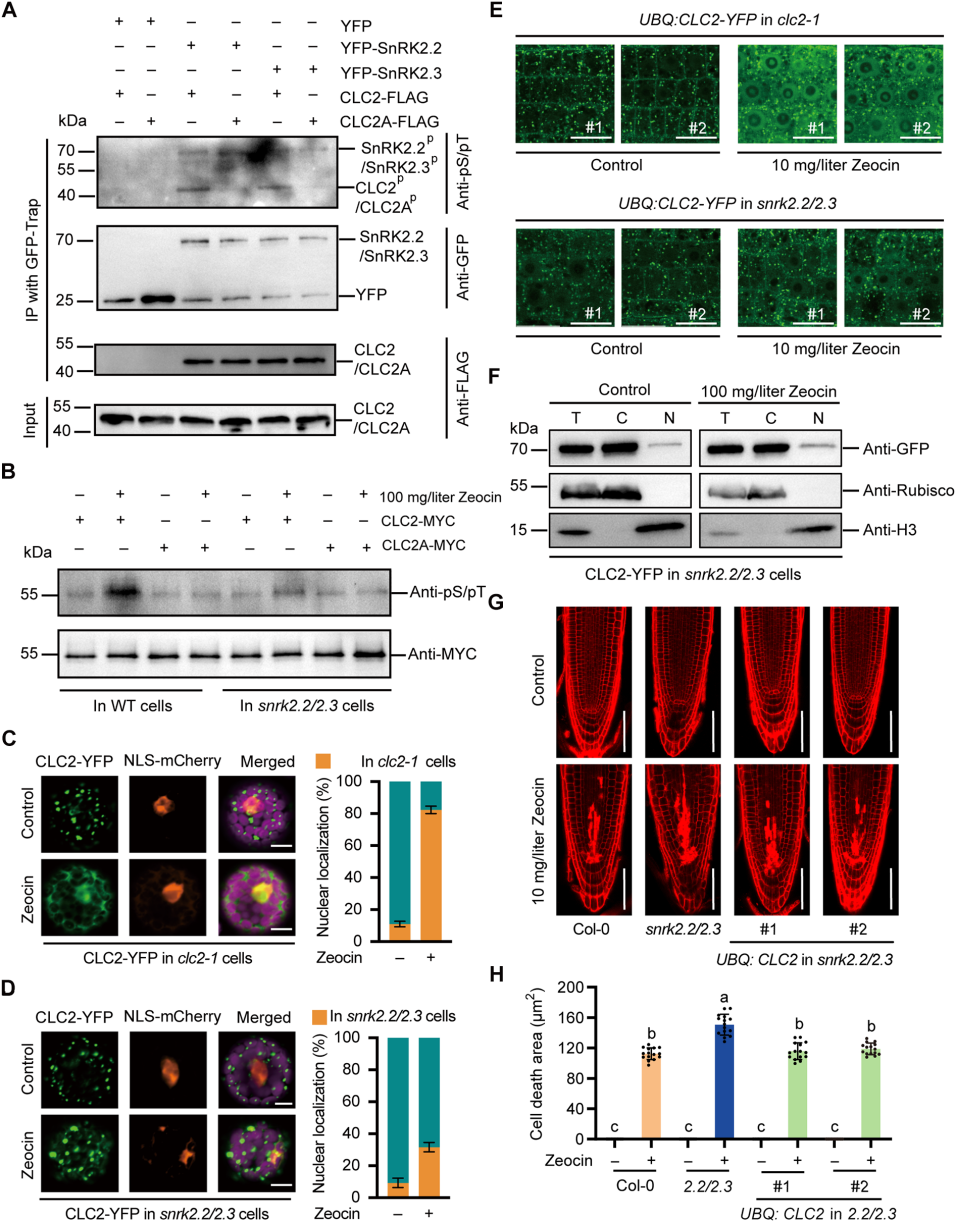
SnRK2s enhances phosphorylation of CLC2. (**A**) In vitro phosphorylation of CLC2 via SnRK2s. The YFP-SnRK2.2 and YFP-SnRK2.3 proteins were immunoprecipitated and incubated with the CLC2-FLAG or CLC2A-FLAG supernatant in kinase buffer for 2 hours. Samples were detected with anti-FLAG, anti-GFP, and anti- phosphoserine/threonine antibodies, respectively. (**B**) In vivo phosphorylation of CLC2 via SnRK2s under DNA damage conditions. CLC2-MYC and CLC2A-MYC were expressed in wild-type or *snrk2.2/2.3* protoplasts with or without Zeocin for 24 hours. The results were detected with anti-GFP and anti-phosphoserine/threonine antibodies, respectively. (**C** and **D**) Localization of CLC2-YFP in *clc2-1* (C) and *snrk2.2/2.3* (D) cells with and without DNA damage. Scale bars, 10 μm. Percentages (means ± SD; *n* = 100) of cells with and without nucleus localization are from three independent experiments. (**E**) The localization of CLC2-YFP in intact *clc2-1* (E) and *snrk2.2/2.3* roots. Five-day-old seedlings are incubated in medium with or without Zeocin for 12 hours before recorded. The images are representative results from three biologically independent experiments. Scale bars, 15 μm. (**F**) The distribution of CLC2-YFP in *snrk2.2/2.3* cells. The vector expressing CLC2-YFP was incubated with or without Zeocin for 24 hours. The CLC2-YFP was detected by an anti-GFP antibody. Anti-Rubisco and anti-H3 antibodies were used for marking the cytoplasmic and nucleus fractions, respectively. (**G** and **H**) The effect of *CLC2* overexpression on cell death in root meristems of *snrk2.2/snrk2.3* mutant. (G) Seeds were sown on ^1^/_2_ MS medium for 5 days before subjected with Zeocin for 12 hours. The root meristems were observed using PI staining. Three biologically independent experiments were conducted, and representative images are shown. Scale bars, 50 μm. (H) Cell death areas were quantified (means ± SD; *n* = 15). Significant differences were indicated using different letters above the columns. The data are means ± SD from triplicated experiments. *P* < 0.001, Tukey’s multiple comparisons test.

Having established that phosphorylation of CLC2 is important for its nuclear localization, we proposed that ABA enhances resistance to DNA damage through SnRK2-mediated phosphorylation of CLC2. To test this, we performed an in vitro phosphorylation assay. The results indicated that both SnRK2.2 and SnRK2.3 are potential kinases for CLC2 (Fig. 5A). Compared to the wild type, mutations at S195 and T197 of CLC2 reduced its phosphorylation by SnRK2s (Fig. 5A). Consistent with these in vitro findings, in vivo experiments showed that phosphorylated CLC2 was notably reduced in the *snrk2.2/2.3* double mutant (Fig. 5B). This indicates that the absence of SnRK2.2 and SnRK2.3 is sufficient to impair CLC2 phosphorylation during the DNA damage response and that S195 and T197 are key phosphorylation sites involved in this process.

On the basis of the data, we further analyzed the relationship between SnRK2s and CLC2. We observed the localization of CLC2 in the *snrk2.2/2.3* double mutant. Results showed that Zeocin treatment triggered more than 80% nuclear shuttling of CLC2 in *clc2-1* cells (Fig. 5C), while only about 30% nuclear shuttling occurred when CLC2 was expressed in the *snrk2.2/2.3* background (Fig. 5D). This suggests a regulatory relationship between SnRK2s and CLC2. To confirm these results in intact plants, we introduced CLC2 fused with YFP into the *clc2-1* and *snrk2.2/2.3* double mutants under the control of the *UBQ10* promoter. In both *clc2-1* and *snrk2.2/2.3* mutants, CLC2 localized to the TGN/EE under normal conditions (Fig. 5E). Zeocin treatment enhanced the nuclear localization of CLC2 in its complementary lines, but in the *snrk2.2/2.3* double mutant, the nuclear shuttling of CLC2 was suppressed under Zeocin stress (Fig. 5E). Our nucleus fractionation assays further showed that the nucleus accumulation of CLC2 was impaired in the *snrk2.2/2.3* mutant under Zeocin treatment (Fig. 5F). These findings are consistent with our hypothesis that SnRK2.2 and SnRK2.3 regulate the localization of CLC2 through its phosphorylation.

Our observations suggested that the nuclear shuttling of CLC2 depends on its phosphorylation by SnRK2.2 and SnRK2.3. Therefore, we hypothesized that overexpression of *CLC2* in the *snrk2.2/2.3* double mutant might improve its resistance to DNA damage. To test this, we examined the cell death in the root meristems of the *snrk2.2/2.3* mutant upon Zeocin stress. Overexpression of *CLC2* significantly reduced cell death under these conditions (Fig. 5, G and H), consistent with the further root growth assay results (fig. S13, A and B). Subsequently, we evaluated the effects of CLC2A and CLC2D on root growth in the *snrk2.2/2.3* double mutant. While *CLC2* overexpression did not notably affect root growth under normal conditions, *CLC2D* significantly mitigated the root sensitivity of the *snrk2.2/2.3* mutant to Zeocin treatment (fig. S14, A and B). These findings provide further evidence of the functional relationship between SnRK2s and CLC2 in Zeocin-induced response.

### CLC2 is essential for precise DSB localization of ADA2b

Having shown that CLC2 is recruited to the nucleus possibly for DNA damage response, the next question is how CLC2 participates in this process. Previous studies have shown that clathrin is monomeric before entering the nucleus, where it interacts with p53 to control the expression of specific genes involved in DNA damage response and apoptosis (*47*, *48*). However, since plants lack a p53 ortholog and instead use the functionally analogous SOG1, the regulatory mechanisms may differ in plants (*55*).

**Fig. 6.**
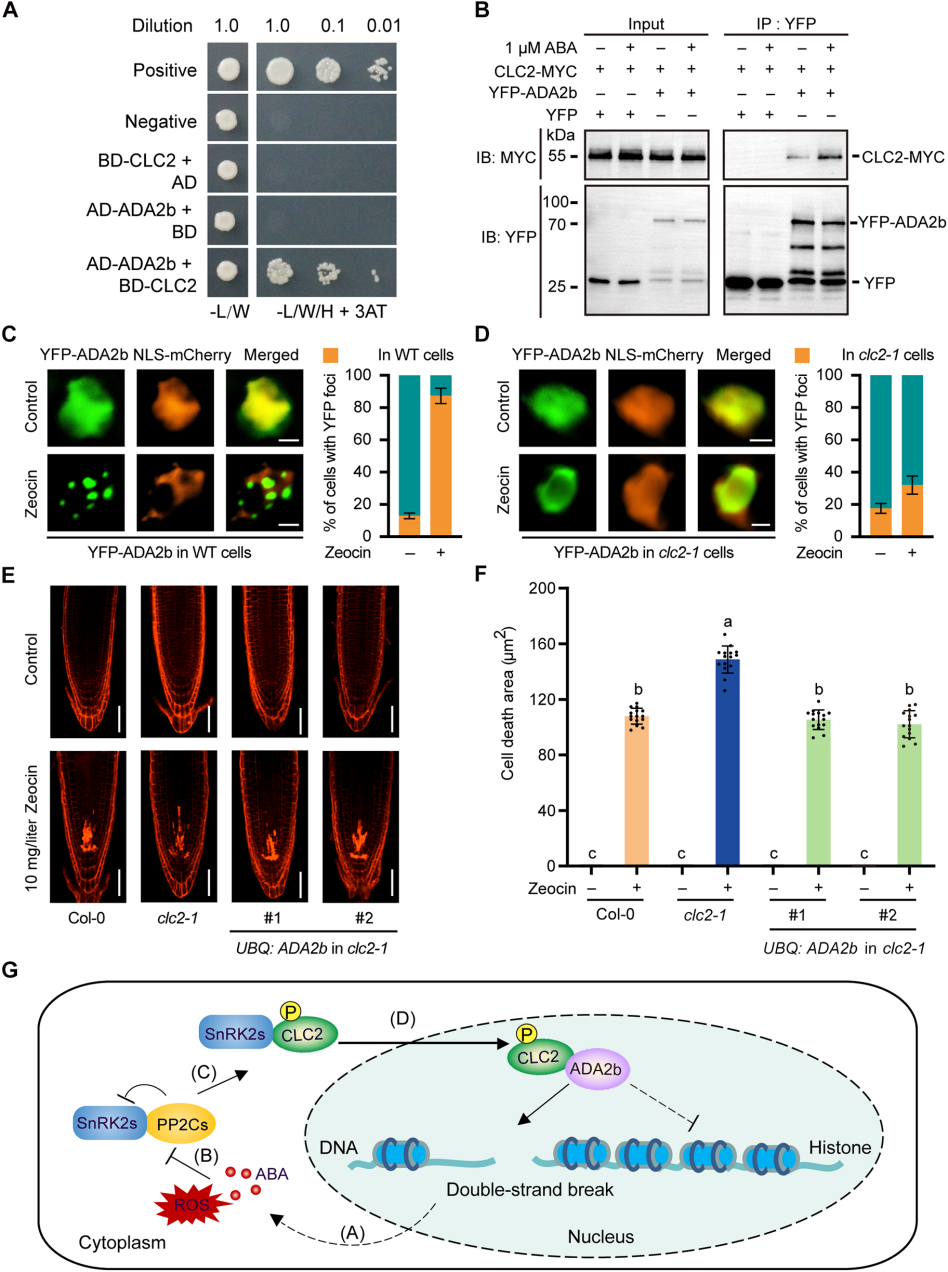
CLC2 is essential for precise DSB localization of ADA2b. (**A**) Interaction between CLC2 (fused with BD) and ADA2b (fused with AD) in yeast two-hybrid assay. The interaction was determined on SD-L-W-H medium containing 30 mM 3-AT. (**B**) Coimmunoprecipitation assay was used to measure the association of CLC2 and ADA2b in plant cells. CLC2-MYC, coexpressed with YFP-ADA2b or free YFP (negative control), was treated with ABA for 12 hours. Total proteins were purified using anti-GFP agarose. Both input and IP proteins were subjected with SDS-PAGE, and the signals were detected using anti-GFP and anti-MYC antibodies, respectively. (**C** and **D**) Subcellar localization of YFP-ADA2b in wild-type (C) and *CLC2* mutant (D) cells with and without DNA damage. Representative images of YFP (green), mCherry (orange), and merged signals are shown in the left-hand graphs. Scale bars, 5 μm. The percentage of cells with (orange) and without (green) YFP foci signals are shown as means ± SD (*n* = 100) from three independent experiments in the right-hand graphs. (**E** and **F**) Cell death area of *YFP-ADA2b* overexpression lines on *clc2-1* mutant under normal and DNA damage conditions. Five-day-old seedlings were transferred to Zeocin for 12 hours. Three biologically independent experiments were conducted, and the representative results stained by PI are shown in (E). Scale bars, 1 cm. The cell death area was presented as means ± SD (*n* = 15) in (F). Letters above the columns indicate the significant differences. *P* < 0.001, Dunnett’s multiple comparisons test. (**G**) A proposed model for ABA-mediated DNA damage response in plant cells. DNA damage up-regulated the ABA and ROS levels in cells, which activates SnRK2s in the ABA signaling pathway for the phosphorylation and nuclear recruitment of CLC2. CLC2 interacted with ADA2b in the nucleus and possibly regulated its chromatin disassociation for further recruitment at DSBs.

To elucidate the role of CLC2 in DNA damage response, we performed a yeast two-hybrid assay to screen its interactor in this process, with the previously reported DNA stress associated factors SMC5, ADA2b, CDC5, IDN2, SWI3B, RAD51, SOG1, and GCN5 included (*12*, *55*, *56*). The results showed that only CLC2 (Fig. 6A) and CLC2D (fig. S15A) interacted with ADA2b. To confirm this interaction, we conducted an in vitro pull-down assay, which showed that GST-tagged CLC2 directly bound to ADA2b-FLAG (fig. S15B), indicating a direct interaction between the two proteins. To verify this interaction in vivo, CLC2 was tagged with MYC and precipitated with YFP-ADA2b but not YFP in plant cells, and the interaction was enhanced upon ABA treatment (Fig. 6B). Moreover, we used a BiFC experiment to validate the interaction, which showed that nYFP-ADA2b complemented with CLC2-cYFP, indicating an interaction between them in vivo (fig. S15C).

We previously reported that ADA2b colocalized with γ-H2AX at DSBs to recruit the SMC5/6 complex in response to DNA damage (*11*). Given that the SMC5/6 complex has been implicated in HR (*8*, *57*) and that HR-related genes are up-regulated in ADA2b mutants (*11*), we further investigated the expression of HR-related genes in CLC2 mutants. Our results showed that the loss of *CLC2* also influenced the expression of these genes (fig. S16). Notably, Zeocin (fig. S16A) and MMS (fig. S16B) treatment exacerbated this up-regulation, suggesting that CLC2 may play a role in modulating the HR repair process in plants. To further investigate the relationship between CLC2 and ADA2b, we expressed CLC2-YFP in wild-type and *ada2b-3* protoplasts. We were unable to detect the DSB localization of CLC2, and the absence of ADA2b did not affect the nuclear localization of CLC2 following DNA damage (fig. S17, A and B). Conversely, the DSB localization of YFP-ADA2b was significantly reduced in *clc2-1* protoplasts (Fig. 6, C and D). Since the nuclear localization of CLC2 is partially regulated by the ABA signaling pathway, we examined the impact of ABA signaling on the localization of YFP-ADA2b. The results indicated that the DSB localization of ADA2b was unaffected in the *abi1-2/abi2-2* double mutant (fig. S18A), while the *snrk2.2/2.3* double mutant significantly impaired the DSB localization of ADA2b under DNA damage conditions (fig. S18B). In the absence of CLC2, overexpression of *ADA2b* may increase the pool of free ADA2b, allowing some to localize to damage sites. To test our hypothesis, we first overexpressed *ADA2b* in the wild-type background. The result showed that ADA2b overexpression did not affect root growth under either normal or DNA stress conditions (fig. S19, A and B). However, when ADA2b was overexpressed in the *clc2-1* background, the root meristem response to DNA damage was significantly attenuated (Fig. 6, E and F). Consequently, the root sensitivity of *clc2-1* plants to DNA damage was markedly reduced (fig. S20, A and B). These data collectively suggest that the recruitment of ADA2b to DSBs is dependent on ABA-triggered nuclear shuttling of CLC2.

## DISCUSSION

DNA damage can be induced by various environmental stresses, including drought, high salinity, and osmotic imbalance. ABA, a crucial phytohormone, is up-regulated in response to these abiotic stresses (*17*). ABA enhances plant tolerance by modulating gene expression, protein activity, and ion channel function (*58*). Evidence from public databases indicates that ABA regulates the expression of DNA damage response–related genes to varying degrees (*50*). This differential regulation is likely to play a role in enhancing plant tolerance to environmental stress. Although high concentrations of ABA have been reported to increase DNA damage in plants, and some somatic HR defective mutants exhibit heightened sensitivity to ABA treatment (*32*, *34*), the direct mechanism by which ABA regulates DNA damage response is unknown. In this study, we demonstrated that ABA supplementation significantly improved plant tolerance to DNA damage via the HR pathway, implying that ABA directly mediated DNA damage response.

In addition, Zeocin and MMS treatment increase ABA and ROS levels, activating the ABA signaling pathway in plant cells. This activation not only suggests the involvement of the ABA signaling pathway in DNA damage response but also highlights a positive feedback loop between ABA and ROS. In this loop, ROS may further amplify the ABA response (*59*), enhancing the plant’s ability to cope with DNA damage. MMS and Zeocin primarily induce DSBs, a major form of DNA damage, which severely threatens genome stability (*2*, *3*). This provides further rationale for focusing on DSB repair in our study, as DSBs are critical in maintaining genome integrity.

ABA binds PYR/PYL/RCAR receptors, inhibiting PP2C phosphatases and activating SnRK2 kinases, thereby enhancing plant tolerance to stress (*25*). However, their roles in DNA damage response were previously unknown. Our data revealed that the *PP2C* double mutant (*abi1*-*2/abi2*-*2*) exhibited an enhanced ABA-mediated promotion of Zeocin tolerance, while the *SnRK2* double mutant (*snrk2.2/snrk2.3*) exhibited impaired ABA effects on Zeocin tolerance, indicating that ABA signaling is possibly important for DNA damage response. Although SnRK2.6 is also reported as a key kinase in the ABA pathway (*30*), the double mutant of SnRK2.2 and SnRK2.3 was sufficient to inhibit ABA-enhanced DNA damage tolerance. This may be attributed to the fact that SnRK2.6 is primarily expressed in guard cells and vascular tissues, with minimal expression in roots (*60*, *61*). Together, these findings provide evidence that ABA enhances plant tolerance to DNA damage, potentially through an improvement in the DNA repair efficiency. Further investigation is needed to elucidate the precise mechanisms by which these factors contribute to DNA damage response.

CME is the principal endocytic pathway in mammalian cells, with clathrin dysfunction often associated with diseases such as cancer, neurodegenerative disorders, and immune system disorders (*62*). In plants, clathrin plays a role in stress response like immunity, autophagy (*41*), and heat (*42*), indicating its diverse functions in plant stress resistance. However, its role in DNA damage response remains unclear. Our data indicate that CLC2 is not involved in Zeocin- and MMS-induced endocytosis inhibition, suggesting that it may directly participate in the DNA damage response. We found that SnRK2 kinases interact with CLC2, enhancing its phosphorylation and nuclear localization in response to DNA damaging agent and ABA treatment. CLC2 does not interact with the p53-like protein SOG1 in plants, implying that it may not regulate gene expression during DNA damage response. Our data showed that the TGN/EE localization of CLC2 is reduced upon treatment with either ABA or Zeocin. Double mutation of CLC2 at S195/T197 (CLC2A) prevents this reduction, suggesting that the phosphorylation is important for the nucleus localization of CLC2. In addition, phosphorylated CLC2 does not localize to the nucleus under normal conditions, further suggesting that CLC2 phosphorylation might be involved in its disassociation from clathrin heavy chains, although this mechanism requires further elucidation.

Precise recruitment of repair factors to the DSB lesions is crucial for the efficient DNA repair. Previously, we identified the role of the diRNA/AGO2-associated IDN2-CDC5-ADA2b protein scaffold in recruiting the conserved SMC5/6 complex to DSB sites in the HR repair pathway (*12*). ADA2b, a subunit of the SAGA complex, is dynamically associated with chromosomes under normal conditions and is essential for histone H3 acetylation in eukaryotic cells (*10*, *56*). Upon DNA damage, ADA2b is recruited to DSB lesions (*11*), but the regulatory mechanisms governing the localization of ADA2b remain unclear. In the present work, we further identified CLC2 as an interactor of ADA2b and demonstrated that CLC2 is indispensable for the DSB localization of ADA2b. However, we observed no DSB localization of CLC2 in either protoplasts or intact plants, suggesting that CLC2 might be involved in the chromosomal dissociation of ADA2b before its recruitment to DSB lesions. This mechanism may be analogous to the previously observed regulation of SMC5/6 complex movement to DSB sites by SWI3B, a subunit of the SWI/SNF complex (*9*). Further investigation is needed to verify this possibility.

On the basis of the present study, we propose the following model: Upon DNA damage, ABA and ROS levels are up-regulated, leading to the inhibition of the PP2C-mediated suppression of SnRK2 kinases. This inhibition leads to the activation of SnRK2s and results in the phosphorylation and nuclear accumulation of CLC2. The nuclear-localized CLC2 then binds to ADA2b, facilitating its localization at the damage foci (Fig. 6G). Consequently, pretreatment with ABA before DNA damage activates the DNA damage response system and enhances plant tolerance to DNA damage. Although our study identifies CLC2 as a new factor in the DNA damage response, it is important to note that CLC2 only partially contributes to the ABA-mediated DNA damage response. Given that CLC2 was regulated by hormones such as auxin (*40*), future research should explore whether auxin influences DNA repair through CLC2. In addition, since clathrin light chains exhibit functional redundancy in plants, other light chains might take over the role of CLC2. Therefore, it would also be important to test whether other components of clathrin are involved in this process. Together, our study elucidates an ABA-enhanced DNA damage response pathway with a novel function of CLC2 in maintaining genomic stability. These findings advance our understanding of the plant DNA damage tolerance mechanisms and offer potential strategies for improving plant stress resistance through targeted manipulation of this pathway.

## MATERIALS AND METHODS

### Plant materials and growth conditions

All *Arabidopsis thaliana* seeds used in this study were in a Columbia-0 background. The *abi1*-*2/abi2*-*2* (SALK_072009; SALK_015166C) (*26*), *snrk2.2/2.3* (GABI-Kat 807G04; SALK_096546) (*30*), *clc2*-*1* (SAIL_016049) (*40*), and *ada2b*-*3* (SALK_019407) (*11*) were all described in the previous studies.

Seeds were surface-sterilized [soaking in 75% (v/v) ethanol for 3 min, 10% (v/v) NaClO solution for 4 min, followed by rinsing five times with sterilized water] and placed at 4°C in the dark for stratification for 2 days before sowing on ^1^/_2_ Murashige and Skoog (MS) medium containing 1.5% (w/v) sucrose and 1% (w/v) agar. Seedlings were grown in a greenhouse at 22°C with a 16-hour light/8-hour dark cycle. The light intensity was 180 μmol/m^2^ per s. For DNA damage tolerance assay, 4-day-old seedlings were transferred to ^1^/_2_ MS medium containing indicated ABA (Solarbio, A8060) for 8 hours before treated with Zeocin (15 mg/liter; Solarbio, Z8020). Photographs were taken after treated for 2 days. For DNA damage sensitive assay, seeds were sown on ^1^/_2_ MS medium with Zeocin (15 mg/liter) and grown for 7 days before recording.

### Generation of transgenic plants

Transgenic plants were prepared using the floral dip method with *Agrobacterium tumefaciens* strain GV3101. For construction of overexpression lines, the corresponding coding regions were amplified and inserted into the *pCAMBIA1300*-*221*-*ProUBQ10:YFP* vector and transformed to the indicated backgrounds. To overexpress ADA2b in *clc2*-*1* mutant, the previously described *35S:GFP*-*ADA2b* was used to cross with *clc2*-*1*, and phenotype analysis was conducted using the homozygous offsprings. Functional analyses were performed using homozygous transgenic seedlings with a homozygous transferred DNA mutation background based on genotyping and expression measurements.

### HR efficiency assays

The *I-SceI* endonuclease gene was cloned into *pER10* vector under the control of *XVE* promoter, and then the construct was introduced into a preexisting *IU.GUS* reporter line (*51*). Two independent lines were selected for subsequent HR efficiency assays. For the HR assays, germination was initiated on ^1^/_2_ MS medium. Additional treatment groups were supplemented with 5 μM β-estradiol to induce *I-SceI* expression and/or combined with 0.1 μM ABA to evaluate the effect of ABA on HR efficiency. After 7 days, seedlings were subjected to GUS staining using the GUS Staining Kit (Solarbio, G3061). HR efficiency was quantified by counting the number of GUS-positive cotyledons in each group, with 100 cotyledons randomly selected in each experiment.

### Gene expression analysis

Rosette leaves from different genotypes were used for RNA extraction using the FastPure Universal Plant Total RNA Isolation Kit (Vazyme, RC411-01) according to the manufacturer’s instructions. Reverse transcription was performed using the HiScript III 1st Strand cDNA Synthesis Kit (Vazyme, R312-02). Quantitative reverse transcription polymerase chain reaction was conducted using the Bio-Rad CFX 96 system (C1000 Thermal Cycler) and Bio-Rad CFX Manager software version 3.1. Expression levels were analyzed using the 2-ΔΔCq method.

### ROS detection by DAB and NBT staining

ROS accumulation was assessed using 7-day-old Arabidopsis seedlings treated with Zeocin (50 mg/liter) or MMS (100 mg/liter) in liquid ½ MS medium for 8 hours. After treatment, seedlings were rinsed thoroughly with sterile water and stained using commercial ROS detection kits from Solarbio [3,3′-Diaminobenzidine (DAB) method: G4815; nitro blue tetrazolium (NBT) method: G4816], following the manufacturer’s instructions. For DAB staining, seedlings were incubated in DAB solution in the dark for 30 min, followed by decolorization in clearing solution at 80°C. For NBT staining, a similar procedure was used with NBT staining solution. After decolorization, samples were stored in preservation solution and observed under a phenix MC-DK10U3(C) stereomicroscope.

### Yeast two-hybrid assay

The coding sequences of *CLC2* and *SnRK2s* were cloned into *pGBKT7* and *pGADT7* vectors, respectively. The *pGADT7*-*ADA2b* was previously described. Yeast two-hybrid assays were conducted according to the handbook of Clontech. The transformations were screen using SD/-Leu/-Trp minimal medium (Solarbio, S6110), and the interactions were identified in SD/-Leu/-Trp/-His minimal medium (Solarbio, S4630) supplied with 3-amino-1,2,4-triazole (3-AT).

### Pull-down assay

To confirm the interaction of CLC2-SnRK2s and CLC2-ADA2b in vitro, the coding sequence of CLC2 was cloned into the *pGEX4T*-*1* with a GST tag, and the *SnRK2.2* or *SnRK2.3* was cloned into *pCDFDuet*-*1* with a FLAG tag. The *ADA2b*-*FLAG*-*pCDFDuet*-*1* vector was previously reported. All these recombinant vectors were respectively transformed into *Escherichia coli* BL21 strain for protein expression induction using 0.5 mM isopropyl-β-d-thiogalactopyranoside. Total protein was extracted with the protein extract buffer [50 mM tris-HCl (pH 7.4), 120 mM NaCl, 5% (v/v) glycerol, 0.5% (v/v) NP-40, 1 mM phenylmethylsulfonyl fluoride, and 1 mM β-mercaptoethanol]. The GST-CLC2 and GST control extracts were incubated with GST-Sefinose (TM) Resin 4FF (Sangon Biotech, C600031) for 120 min at 4°C, respectively. Then, the resins were collected for incubation of SnRK2s-FLAG or ADA2b-FLAG extract for 120 min at 4°C. After the incubation, the resins were washed five times using wash buffer [50 mM tris-HCl (pH 7.4), 120 mM NaCl, 5% (v/v) and glycerol] and mixed with protein sample buffer, boiled for 5 min for SDS–polyacrylamide gel electrophoresis (SDS-PAGE) and immunoblots using anti-GST (TransGen Biotech, HT601-01), anti-FLAG antibody (TransGen Biotech, HT201-01), and anti-mouse immunoglobulin G [IgG; Cell Signaling Technology (CST), #7076].

### Coimmunoprecipitation assay

The in vivo interaction of SnRK2s-CLC2 and ADA2b-CLC2 was identified using coimmunoprecipitation. The SnRK2s and ADA2b were cloned into the pBluescript-based *Pro35S:YFP* vector, and CLC2 was amplified and cloned into the pBluescript-based *Pro35S:MYC* vector. *Pro35S:YFP*-*SnRK2s* or *Pro35S:YFP*-*ADA2b* was cotransformed with *Pro35S:CLC2*-*MYC*, respectively. *Pro35S:YFP* was used as a negative control in the assay. After incubation overnight, the protoplasts were collected and incubated with extraction buffer [10 mM tris-HCl (pH 7.4), 100 mM NaCl, 10% (v/v) glycerol, and 0.5% (v/v) NP-40] with protease inhibitor cocktail (Roche, Switzerland). The extractions were then centrifuged at 13,000*g* at 4°C for 20 min, and the supernatants were subjected GFP-Trap resin (AlpaLife, KTSM1301) at 4°C for 2 hours. Then, the resins were collected and rinsed three times with washing buffer [10 mM tris-HCl (pH 7.4), 100 mM NaCl, and 10% (v/v) glycerol]. All samples were resuspended with protein sample buffer and boiled for 5 min for SDS-PAGE and immunoblots using anti-GFP (TransGen Biotech, HT801-01), anti-MYC antibody (TransGen Biotech, HT101-01), and anti-mouse IgG (CST, #7076).

### Fluorescence microscopy

For observation of the localization of CLC2 or ADA2b in protoplasts, CLC2 was cloned into the pBluescript-based *Pro35S:YFP* vector. The *YFP*-*ADA2b* was as described above. The vectors were transformed into protoplasts and incubated 12 hours for protein expression in W5 solution containing with or without Zeocin. NLS-mCherry was described in the previous report and used as a nuclear marker. The results were observed in a Zeiss LSM 800 confocal microscope.

For root meristem observation, 4-day-old seedlings were transferred into ^1^/_2_ MS medium with or without ABA for 8 hours, and then the roots were transferred to another ^1^/_2_ MS medium containing both ABA and Zeocin for another 8 hours. PI (Sangon Biotech, E607306) was used for cell wall staining, and signals were observed under the Leica STELLARIS 5 confocal microscope.

To observe the effect of Zeocin treatment on the endocytosis, 5-day-old seedlings were transferred to Zeocin treatment for 12 hours. Roots were stained with FM4-64 (Invitrogen, F34653) before observation. The signals were detected using the Leica STELLARIS 5 confocal microscope, and the results were statistically analyzed using the ImageJ software.

### Nuclei extraction and fractionation

Nuclei was isolated from protoplasts using nuclei isolation buffer [50 mM tris-HCl (pH 7.4), 200 mM KCl, 250 mM sucrose, 25% (v/v) glycerol, 2 mM EDTA, 2.5 mM MgCl_2_, and 30 mM 2-mercaptoethanol] with 50 μM MG132 (MedChemExpress, HY-13259) and 1× protease inhibitor cocktail (Sigma-Aldrich, P9599). The extracts were centrifuged at 1500*g* for 20 min at 4°C. Then, the supernatants were used for another centrifugation at 13,000*g* for 10 min at 4°C for and saved as the cytosolic fraction; the pellets were rinsed five times with nuclei wash buffer [50 mM tris-HCl (pH 7.4), 25% (v/v) glycerol, and 2.5 mM MgCl_2_] and saved as the nuclear fraction. All samples were subjected with protein sample buffer with 1% (v/v) Triton X-100 and boiled for 5 min for SDS-PAGE and immunoblots using indicated antibodies.

### In vitro phosphorylation assay of CLC2

The phosphorylation assay was performed following the method described previously (*53*). *CLC2* or *CLC2A* was cloned into *pCDFDuet*-*1* with a FLAG tag. The proteins were expressed in *E. coli BL21* strain. YFP-tagged SnRK2.2 and SnRK2.3 proteins were isolated through immunoprecipitation from *UBQ:YFP*-*SnRK2.2* and *UBQ:YFP*-*SnRK2.3* transgenic plants that were treated with 100 μM ABA for 60 min.

In vitro kinase assays were conducted by incubating the immunoprecipitated YFP-SnRK2.2 and YFP-SnRK2.3 with the CLC2-FLAG or CLC2A-FLAG supernatant in kinase buffer [10 mM tris-HCl (pH 7.4), 10 mM MgCl_2_, 0.5 mM dithiothreitol, 2 mM MnCl_2_, 50 mM adenosine triphosphate, and PhosSTOP phosphatase inhibitor cocktail] for 1 hour at room temperature. Samples were subjected with SDS-PAGE and and immunoblots using anti-FLAG (TransGen, HT201-01), anti-GFP (TransGen, HT801-01), and anti-phosphoserine/threonine antibody (ECM Biosciences, PP2551).

### Measurement of ABA content by high-performance liquid chromatography–tandem mass spectrometry

The quantification of ABA was previously described (*63*). Six-day-old seedlings were treated with Zeocin (10 mg/liter) for 1 hour. One hundred milligrams of seedlings was ground into powder for extraction with 1 ml of 80% (v/v) methanol with 3-pg internal standards [with 250-pg internal standards (^2^H_6_-ABA for ABA and ABA-GE quantification and ^2^H_6_-SA for ^2^H_6_-ABA quantification)] and extracted twice at 4°C. The samples were centrifugated at 4°C, 15000*g*, 10 min for supernatant collection and dried with nitrogen gas flow. Then, the pellet was dissolved in 300 μl of 30% (v/v) methanol and centrifugated at 4°C, 15000*g*, 10 min again. The samples were passed through a 0.22-μm membrane filter for ABA and ABA-GE quantification according to a previously described method (*64*, *65*).

### Statistical analysis

Significant differences among different treatments and genotypes were analyzed by two-way analysis of variance (ANOVA) test. Details of statistical analyses are provided in the figure legends.

### Accession numbers

The sequences used in this work were obtained from the Arabidopsis Genome Initiative (https://arabidopsis.org/). The accession numbers were *CLC2* (AT2G40060), *SnRK2.2* (AT3G50500), *SnRK2.3* (AT5G66880), *SnRK2.6* (AT4G33950), *ABI1* (AT4G26080), *ABI2* (AT5G57050), *ADA2b* (AT4G16420), *KU70* (AT1G16970), *KU80* (AT1G48050), *XRCC4* (AT3G23100), *BRCA1* (AT4G21070), *RAD51* (AT5G20850), *PARP2* (AT4G02390), and *ACTIN2* (AT3G18780).
