# Supplementary material for: Abscisic acid enhances DNA damage response through the nuclear shuttling of clathrin light chain 2 in plant cells
Source: Sci Adv. 2025 Jun 13;11(24):eadt2842. doi: 10.1126/sciadv.adt2842 (PMC12164954; doi:10.1126/sciadv.adt2842)
Supplement: Supplementary file 1 — Figs. S1 to S20 Table S1 [file sciadv.adt2842_sm.pdf]

Science Advances
AAAS

Supplementary Materials for

Abscisic acid enhances DNA damage response through the nuclear shuttling of clathrin light chain 2 in plant cells

Jieming Jiang *et al.*

Corresponding author: Jieming Jiang, jiangjieming@scnu.edu.cn; Chao Wang, wangc@usx.edu.cn; Chengwei Yang, yangchw@scnu.edu.cn

Sci. Adv. **11**, eadt2842 (2025)
DOI: 10.1126/sciadv.adt2842

This PDF file includes:

Figs. S1 to S20
Table S1

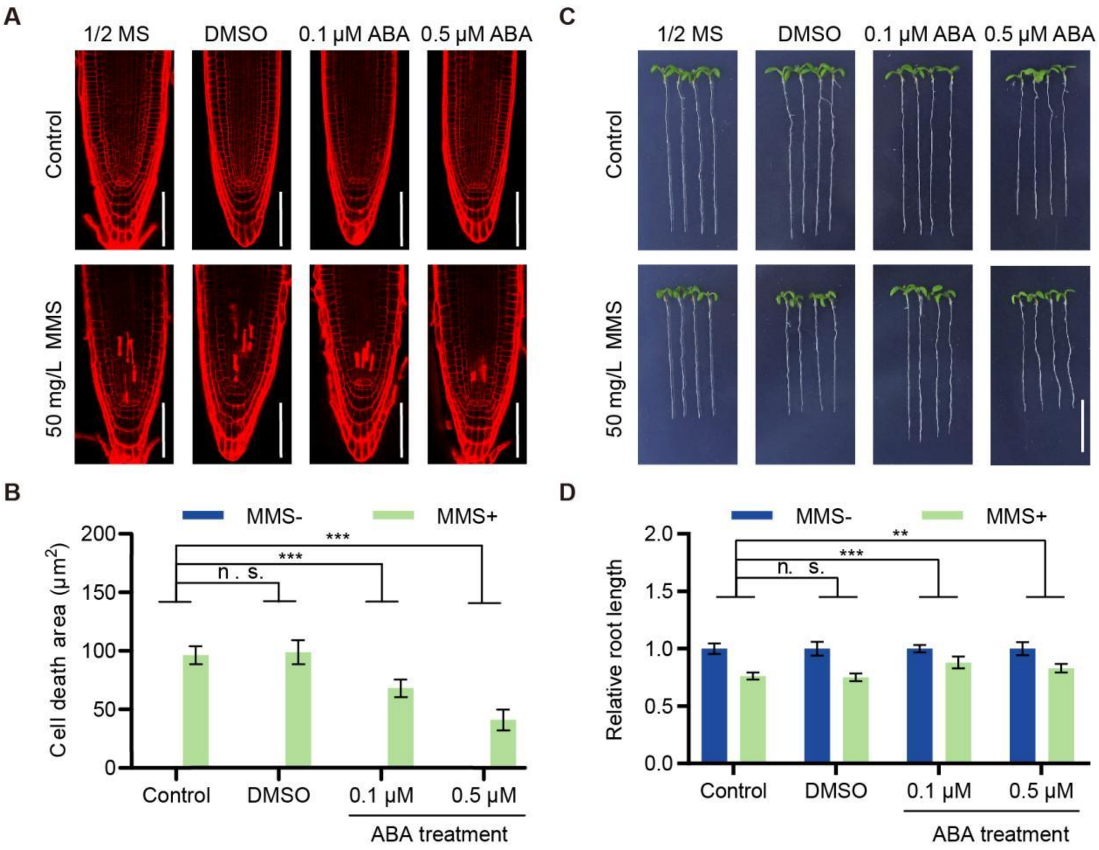

Figure S1

Fig. S1. Effects of ABA on root development upon DNA damage in wild type seedlings. (**A**) The 5-day-old vertically grown seedlings were transferred to medium with or with 0.1 μM ABA for 8 h, and then further incubated in medium with or without 0.1 μM ABA and 50 mg/L MMS for 12 h. DMSO was used as negative controls. The results in their root meristems were observed using PI staining; bars=50 μm. (**B**) Cell death areas were analyzed by ImageJ software. Statistical data with significant differences (means ± SD; n=15) are shown using two-way ANOVA analysis between the control and treatments (Dunnett's multiple comparisons test). ***p<0.001, n.s., no significance. **(C)** Five-day-old seedlings were grown vertically and then transferred to 1/2 MS medium containing or lacking the indicated concentrations of ABA for 8 hours. The seedlings were subsequently moved to fresh medium with or without 50 mg/L MMS and incubated for an additional 48 hours. DMSO was used as the negative control. Representative images from three independent biological replicates are shown. **(D)** Root lengths were measured using ImageJ, and data are presented as the mean ± SD from 15 roots. Significant differences between control and treatment groups were assessed using a two-way ANOVA followed by Dunnett's multiple comparisons test. ***p<0.0001, **p<0.01, n.s., no significance. Scale bar=1 cm.

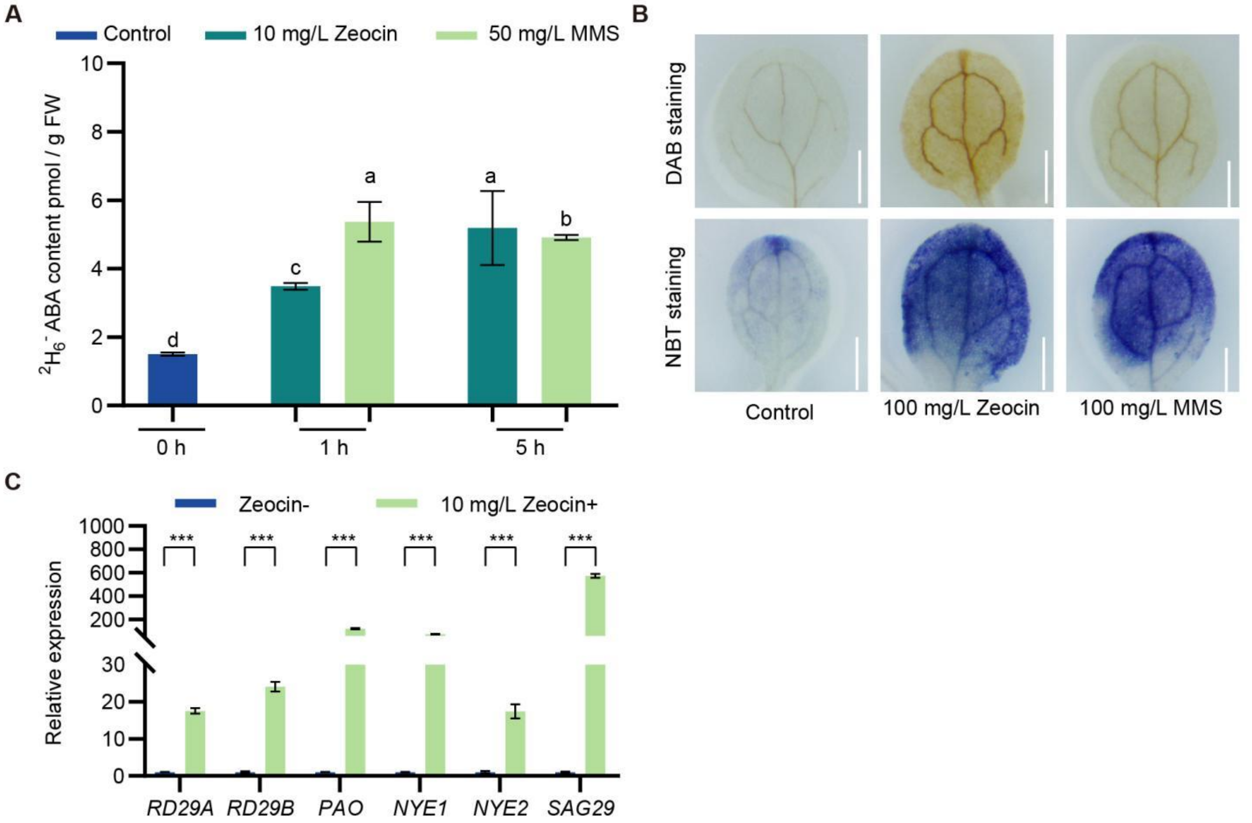

Figure S2

Fig. S2. Effects of DNA damage treatment in ABA and ROS responses. (A) 6-day-old seedings were used for measurement of ABA content after treatment with 10 mg/L Zeocin or 50 mg/L MMS for the indicated time. The data are means ± SD from triplicated experiments. Letters marked above are indication for the significant differences among the columns (Tukey's multiple comparisons test), $p<0.001$. **(B)** 5-day-old seedings were used for DNA damage treatment for the 12 h before they were subjected to further DAB and NBT staining; bars=0.5 mm. **(C)** Expression of ABA responsive genes were detected in 5-day-old seedings after treated with 10 mg/L Zeocin for 12 h via quantitative RT-PCR. The data are means±SD from triplicated experiments. ***$p<0.001$, Student's *t*-test.

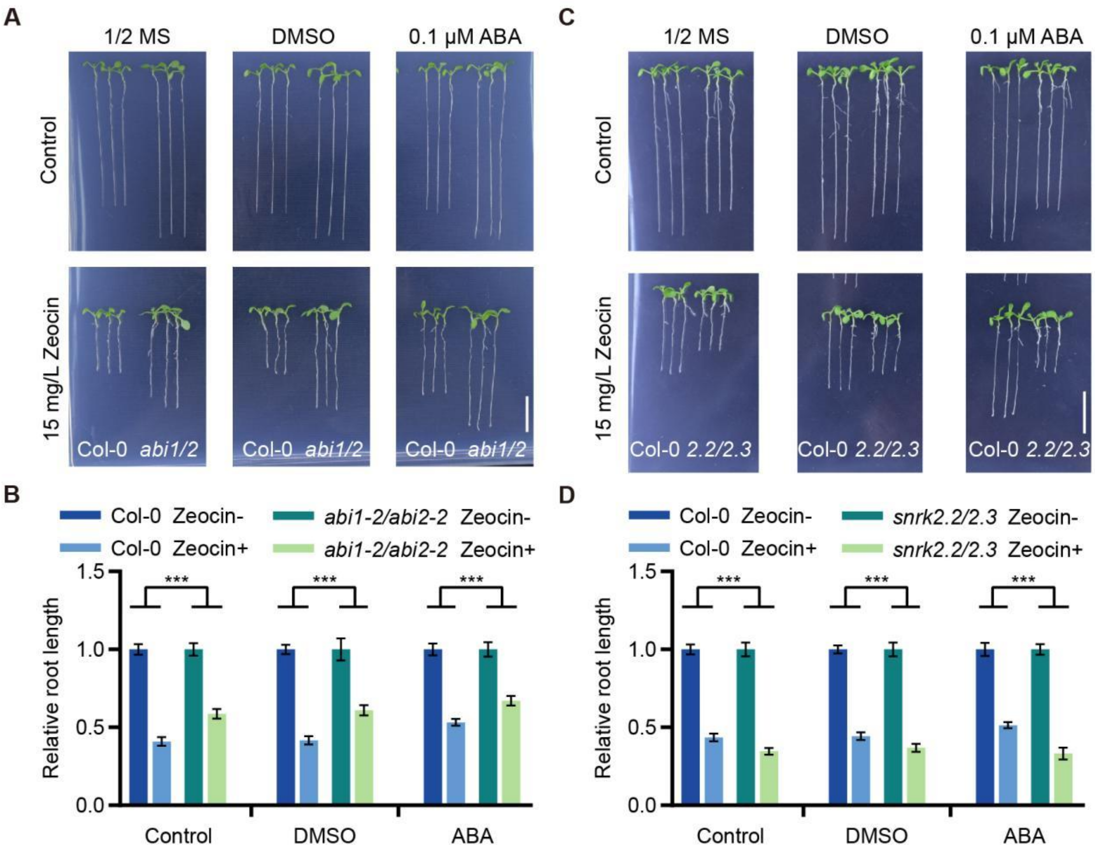

Figure S3

Fig. S3. ABA signaling pathway is involved in DNA damage response. (A, B) Detection of ABA mediated DNA repair in 5-day-old abi1-2/abi2-2 double mutant plants. The seedlings were transferred to 1/2 MS medium with or without 0.1 µM ABA for 8 h, then the pretreated seedlings were transferred to medium with or without 0.1 µM ABA and 15 mg/L Zeocin for another 48 h. Images shown in (A) are representative results from three biologically independent experiments; bar=1cm. The root lengths were quantified using ImageJ and showed in (B). The data are mean±SD from 15 roots with significant differences between Col-0 and mutant seedlings determined using two-way ANOVA test (Dunnett's multiple comparisons test). ***p<0.001. **(C, D)** Detection of ABA mediated DNA repair in 5-day-old snrk2.2/snrk2.3 double mutant plants. The seedlings were transferred to 1/2 MS medium with or without 0.1 µM ABA for 8 h. Then were further transferred to medium with or without 0.1 µM ABA and 15 mg/L Zeocin for another 48 h. Representative images from three biologically independent experiments are shown in (C); bar=1cm. Quantitative data from 15 roots were shown in (D) using ImageJ software. Significant differences between Col-0 and mutant seedlings were determined via two-way ANOVA test (Dunnett's multiple comparisons test). ***p<0.001.

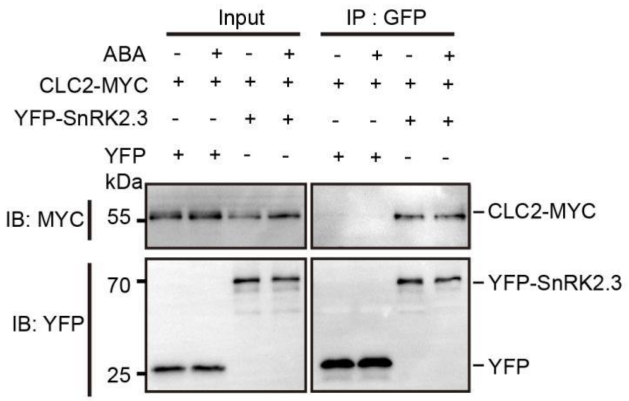

Figure S4

Fig. S4. The association of SnRK2s and CLC2 were measured using co-immunoprecipitation assay in plant cells. CLC2-MYC was co-expressed with YFP tagged SnRK2s or free YFP (negative control) in protoplasts. Immunoprecipitations were conducted using anti-GFP agarose and total protein. The input and IP protein signals were obtained using anti-GFP and anti-MYC antibodies, respectively.

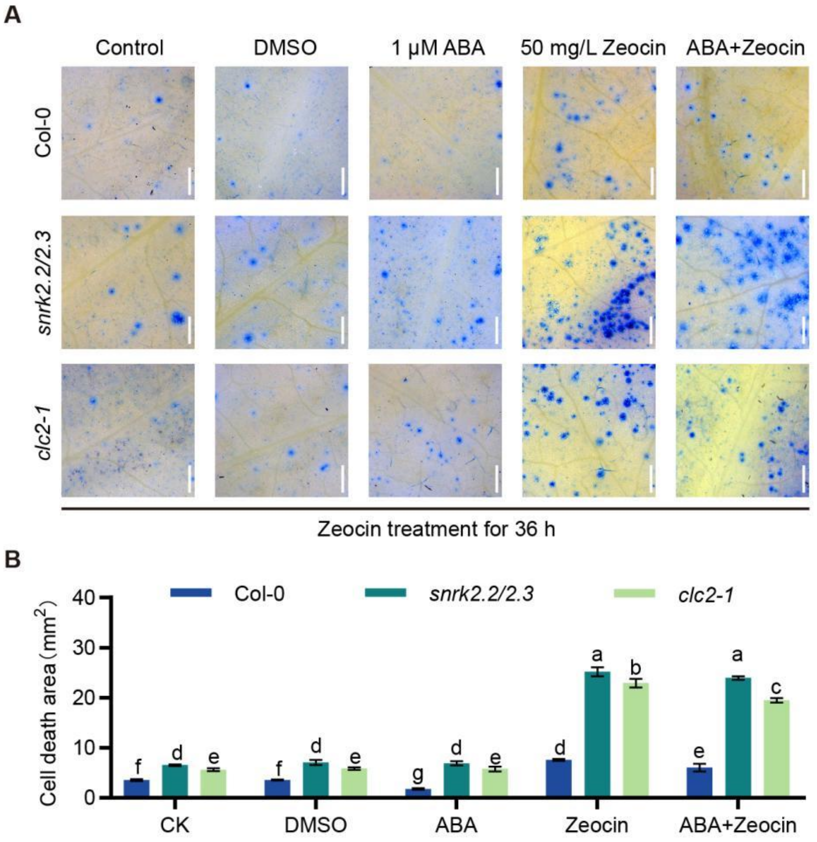

Figure S5

Fig. S5. Detection of *clc2-1* mutant in ABA-mediated DNA damage tolerance. 4-week-old indicated seedlings were used for ABA treatment for 12 h, followed by the treatment of 50 mg/L Zeocin for 36 h before conducting trypan blue assay. The images in (A) are representative of recorded by stereomicroscope; bars=0.5 mm. The cell death areas were quantified using ImageJ and showed in (B). The data are mean ± SD from 3 leaves with significant differences between Col-0 and mutant seedlings determined using one-way ANOVA test (Tukey's multiple comparisons test). p<0.001.

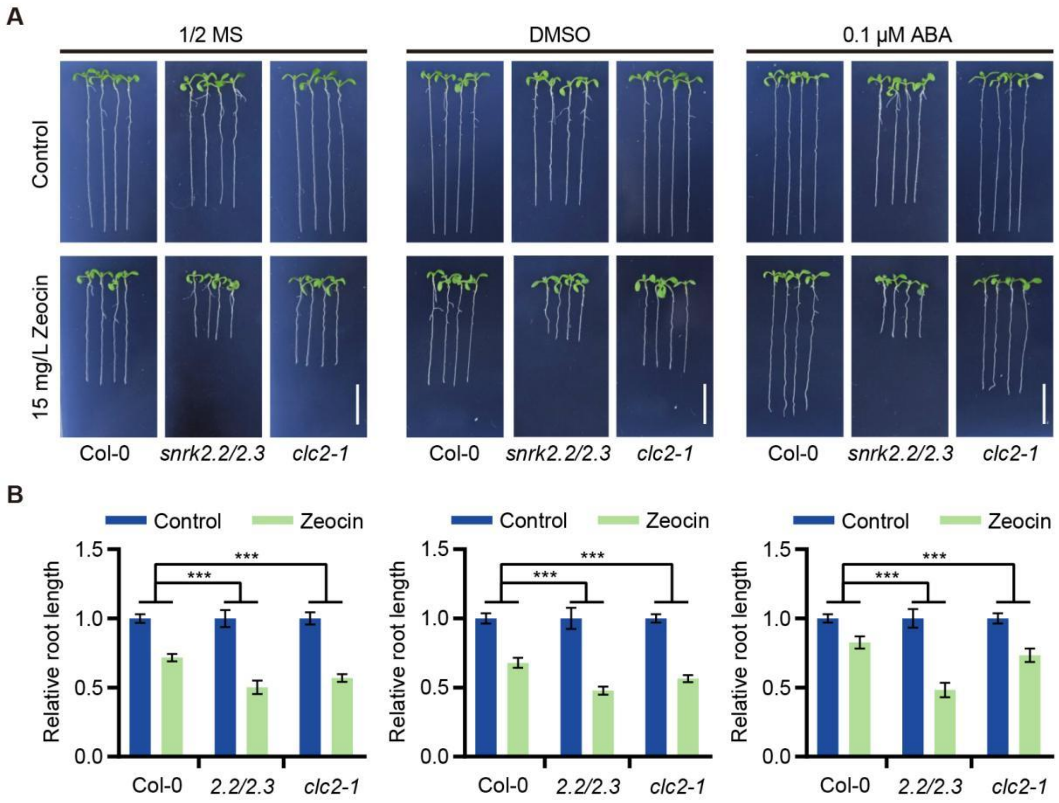

Figure S6

Fig. S6. Detection of ABA mediated DNA damage tolerance in *clc2-1* mutant plants. The seedlings were grown on vertical 1/2 MS medium for 5 days before transferred to 1/2 MS medium containing 0.1 µM ABA or DMSO for 8 h. Then the seedlings were transferred to 1/2 MS medium with DMSO+15 µg/mL Zeocin or 0.1 µM ABA+15 µg/mL Zeocin and incubated for another 48 h. Images shown in (A) were representative results from three biologically independent experiments; bars=1cm. Quantitative data (mean ± SD) of root length (n=15) are shown in (B) using ImageJ software. Significant differences among different genotypes were calculated using two-way ANOVA test (Dunnett's multiple comparisons test). ***p<0.001.

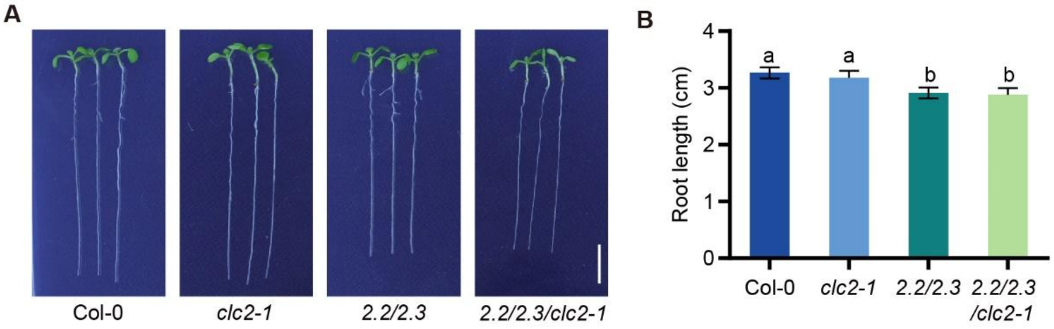

Figure S7

Fig. S7. Analysis of the genetic relationship between *SnRK2s* and *CLC2*. The seedlings were vertically grown in 1/2 MS medium for 7 days before recorded. Representative results from three biologically independent experiments are shown in (A); bar=1 cm. Root length calculated using ImageJ software are shown in (B). Data are presented as the mean±SD (n=15) and the significant differences among different genotypes were calculated using one-way ANOVA test (Tukey's multiple comparisons test). Letters marked above are indication for the significant differences among the columns. $p<0.001$.

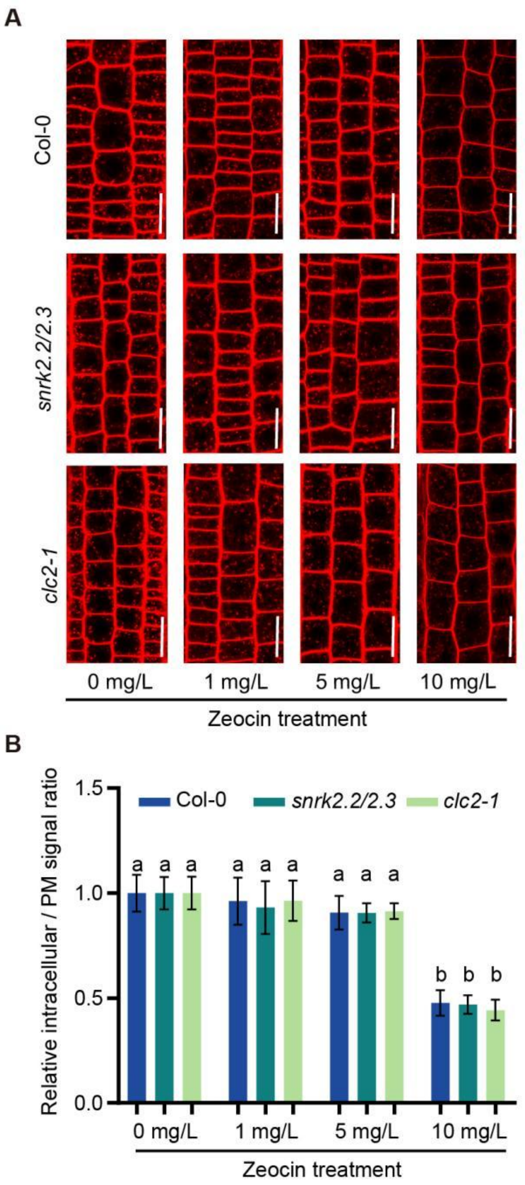

Figure S8

Fig. S8. The effect of DNA damage on the endocytosis in the *clc2-1* and *snrk2.2/2.3* mutant. The seedlings were grown vertically for 5 days before treated with indicated Zeocin and 2 μM FM4-64 for 12 h. (**A**) Representative images from three biologically independent experiments are shown; bars=50 μm. (**B**) The quantitative data (mean ± SD from 15 roots) are shown. Significant differences between the control and treatments were analyzed using one-way ANOVA test (Tukey's multiple comparisons test). Letters marked above are indication for the significant differences among the columns. $p<0.001$.

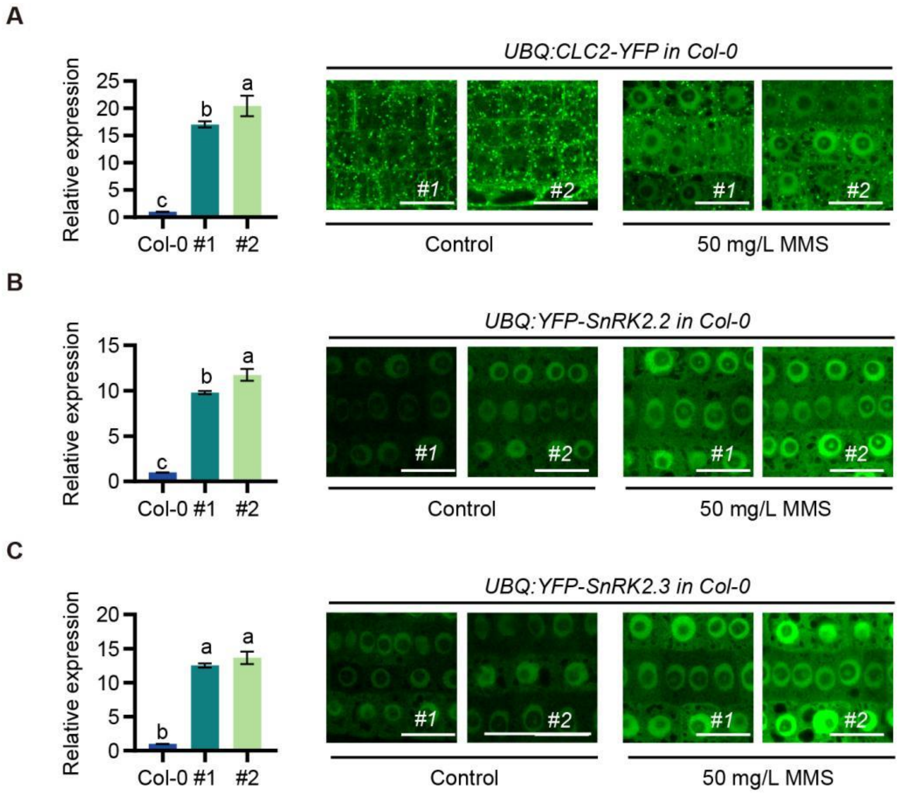

Figure S9

Fig. S9. The localization of CLC2-YFP and YFP-SnRK2s in intact roots of transgenic wild type seedlings. (A) Representative images of CLC2-YFP in wild type seedlings were shown. **(B)** Subcellular localization of YFP-SnRK2.2 in wild type seedlings with or without DNA stress. **(C)** Subcellular localization of YFP-SnRK2.3 in wild type roots with or without DNA stress. Two lines with similar expression levels were selected for the experiments. Five-day-old vertically grown seedlings were incubated in medium with or without 50 mg/L MMS for 12 h before recorded. Representative results from three independent experiments are shown; bars=15 μm. Significant differences between the columns were analyzed using one-way ANOVA test (Tukey's multiple comparisons test). Letters above are indication for the significant differences among the columns. p<0.001.

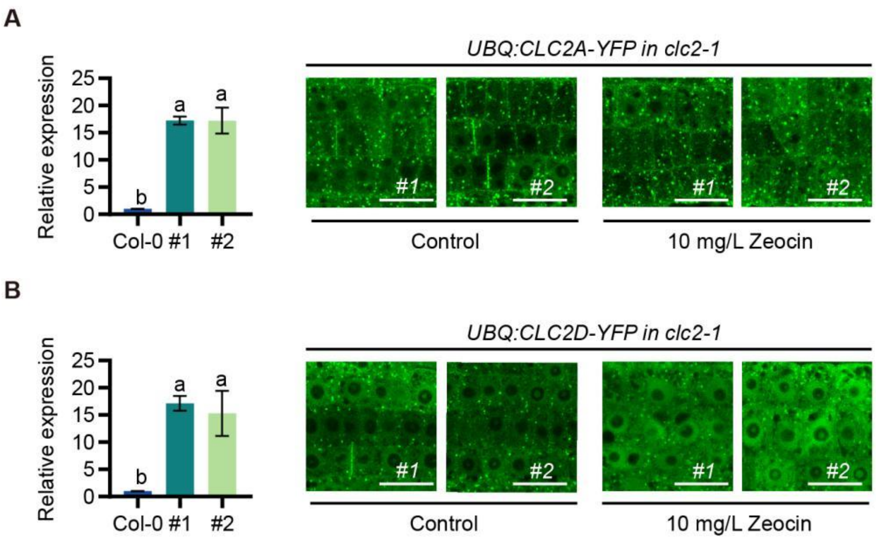

Figure S10

Fig. S10. The localization of CLC2A-YFP and CLC2D-YFP in intact roots of transgenic complementation seedlings. (A) Subcellular localization of CLC2A-YFP in wild type seedlings with or without Zeocin treatment. **(B)** Subcellular localization of CLC2D-YFP in wild type roots with or without Zeocin treatment. Two lines with similar expression levels were selected for the experiments. Five-day-old vertically grown seedlings were incubated in medium with or without 10 mg/L Zeocin for 12 h before recorded. Representative results from three independent experiments are shown; bars=15 μm. One-way ANOVA test (Tukey's multiple comparisons test) was used to analyze the significant differences between the columns, which is indicated using different letters above the columns. $p<0.001$.

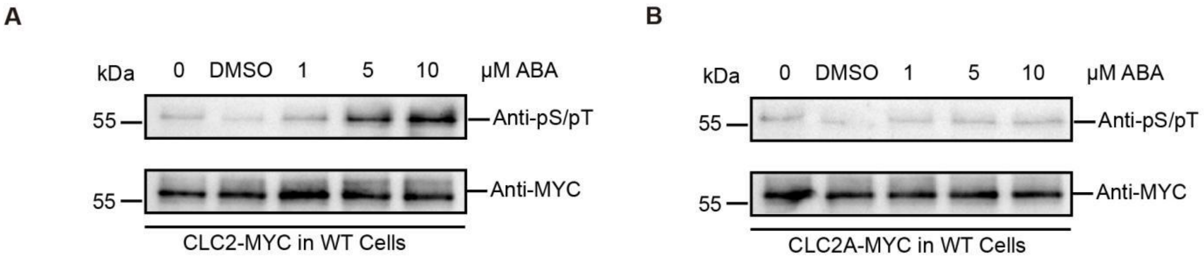

Figure S11

Fig. S11. Phosphorylation levels of CLC2-MYC and CLC2A-MYC upon DNA damage. **(A)** Phosphorylation levels of CLC2-MYC in protoplasts treated with indicated ABA for 24 h. **(B)** Protoplasts expressing CLC2A-MYC were incubated with or without indicated ABA for 24 h before phosphorylation detection. DMSO was used as negative controls.

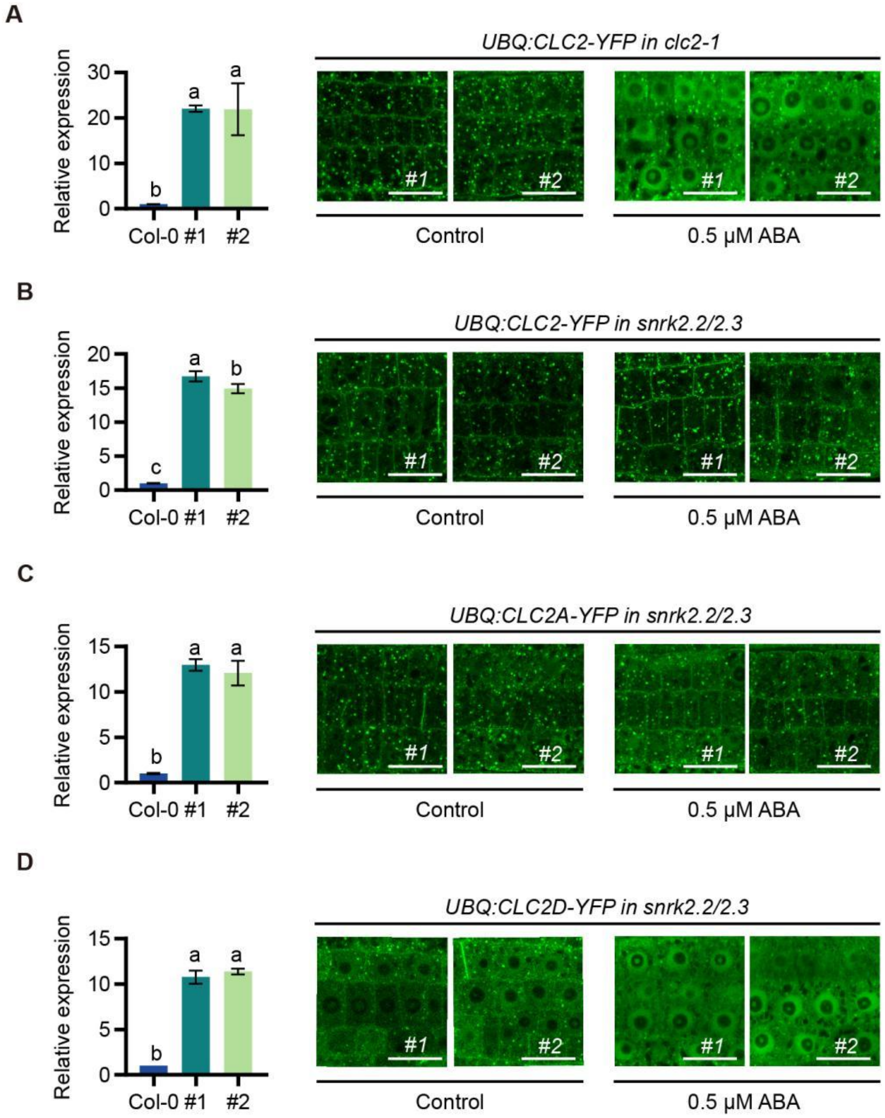

Figure S12

Fig. S12. The localization of CLC2-YFP in intact roots of transgenic seedlings under the treatment of ABA. (A) Subcellular localization of CLC2-YFP in *clc2-1* seedlings with or without ABA treatment. **(B)** Subcellular localization of CLC2-YFP in *snrk2.2/2.3* seedlings with or without ABA treatment. **(C)** Subcellular localization of CLC2A-YFP in *snrk2.2/2.3* seedlings with or without ABA treatment. **(D)** Subcellular localization of CLC2D-YFP in *snrk2.2/2.3* seedlings with or without ABA treatment. Two lines with similar expression levels were selected for the experiments. Five-day-old vertically grown seedlings were incubated in medium with or without 0.5 μM ABA for 12 h before recorded. Representative results from three independent experiments are shown; bars=15 μm. Significant differences between the columns were analyzed using one-way ANOVA test (Tukey's multiple comparisons test). Letters above are indication for the significant differences among the columns. $p<0.001$.

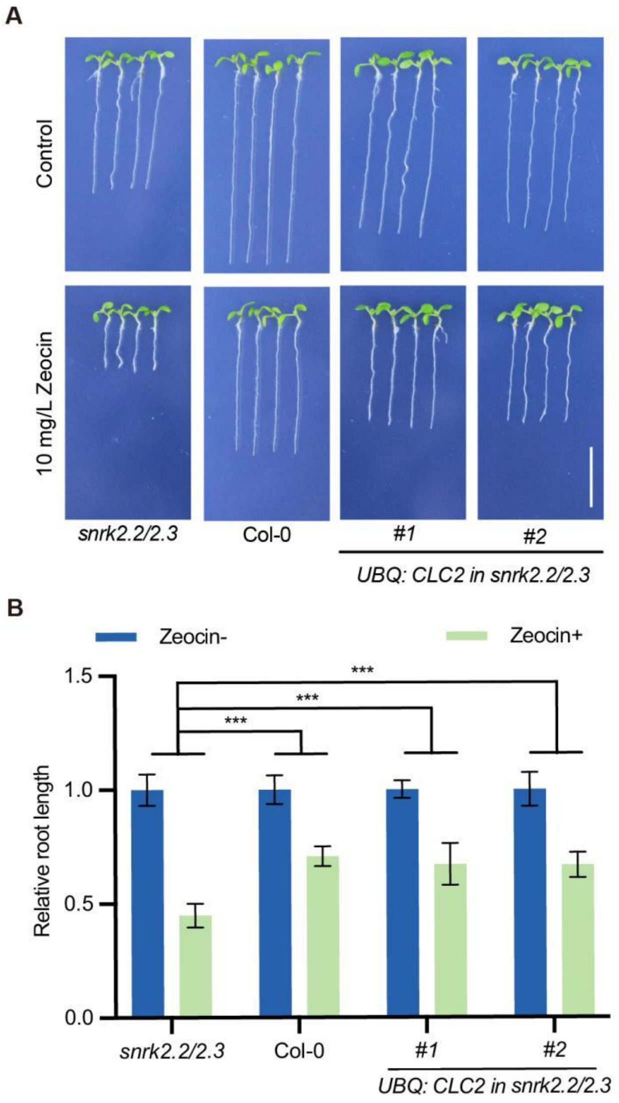

Figure S13

Fig. S13. Phenotypes of *CLC2-YFP* overexpression on *snrk2.2/2.3* mutant under DNA damage. Seedlings were vertically grown in 1/2 MS medium with 10 mg/L Zeocin for 7 days before recorded. The results were from three biologically independent experiments are shown in (A); bar=1 cm. Root length calculated using ImageJ software are shown in (B). Data are presented as the mean±SD (n=15) and the significant differences among different genotypes were calculated using two-way ANOVA test (Dunnett's multiple comparisons test). ***$p<0.001$.

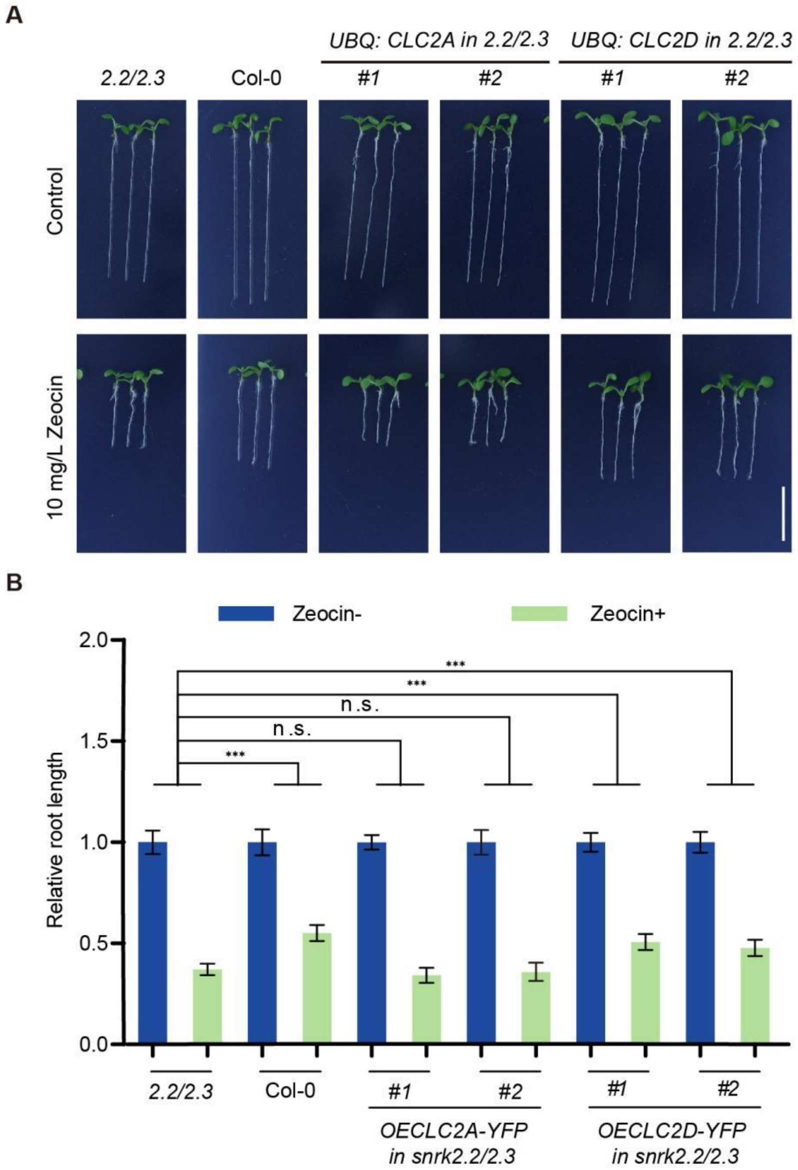

Figure S14

Fig. S14. Phenotypes of CLC2A and CLC2D supplementary *snrk2.2/2.3* lines under DNA damage. Seedlings were grown in 1/2 MS medium with 10 mg/L Zeocin for 7 days before recording. Representative images from three biologically independent experiments are shown in (A); bar=1 cm. Root length was calculated using ImageJ and quantitative data in (B) are presented as the mean±SD (n=15). Significant differences among different genotypes were calculated using two-way ANOVA test (Dunnett's multiple comparisons test). ***p<0.001.

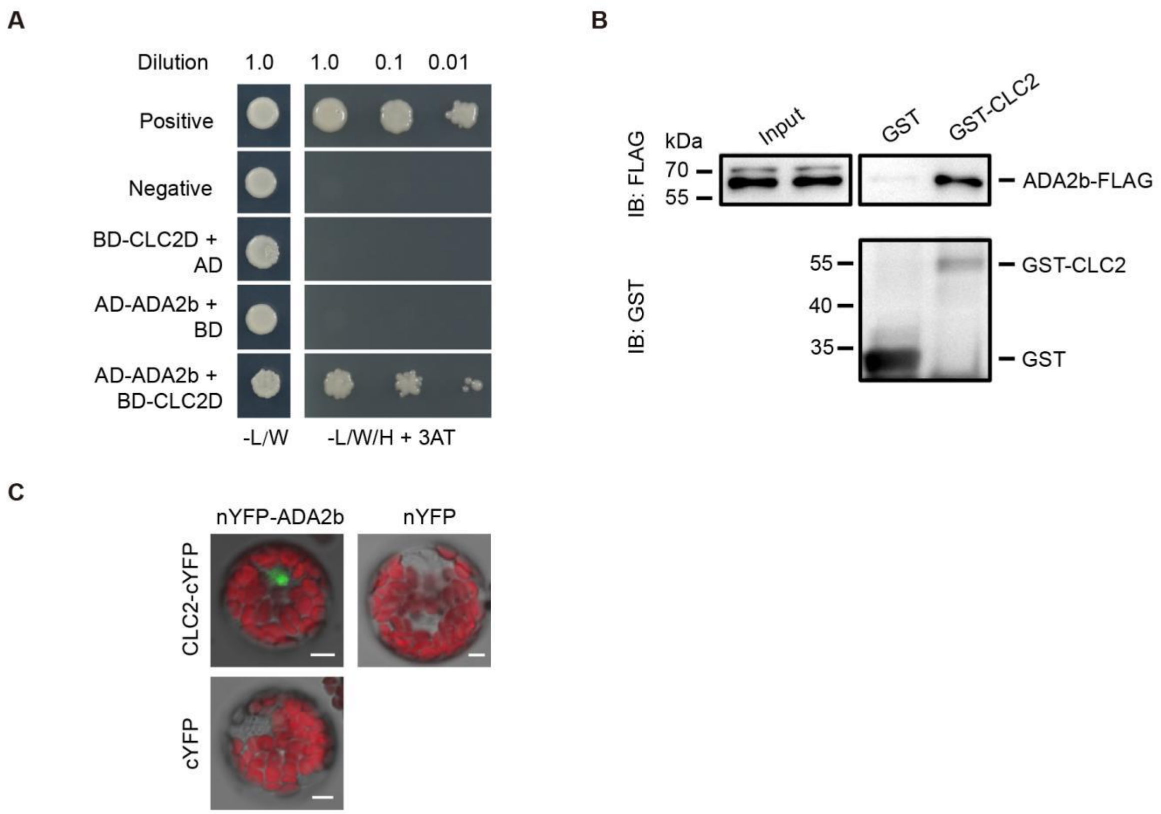

Figure S15

Fig. S15. The *in vitro* and *in vivo* interaction of CLC2 and ADA2b. (**A**) The interaction between ADA2b and CLC2D was detected using yeast two-hybrid assay. ADA2b was fused with AD and CLC2D was fused with BD. The interaction was determined on SD-L-W-H medium containing 30 mM 3-AT. (**B**) GST pull-down assay was used to detected the interaction of ADA2b and CLC2 *in vitro*. ADA2b was fused with FLAG tag while CLC2 was fused with GST tag. ADA2b precipitated with GST-CLC2 and free GST (negative control) were detected using an anti-FLAG antibody. (**C**) BiFC assay used to detected the interaction of CLC2 and ADA2b in plant cells. ClC2 was fused with C-terminal of YFP (cYFP) and ADA2b with N-terminal of YFP (nYFP). The vectors were cotransformed into the protoplasts and incubated for 24 h before YFP signals were observed. bars=10 μm.

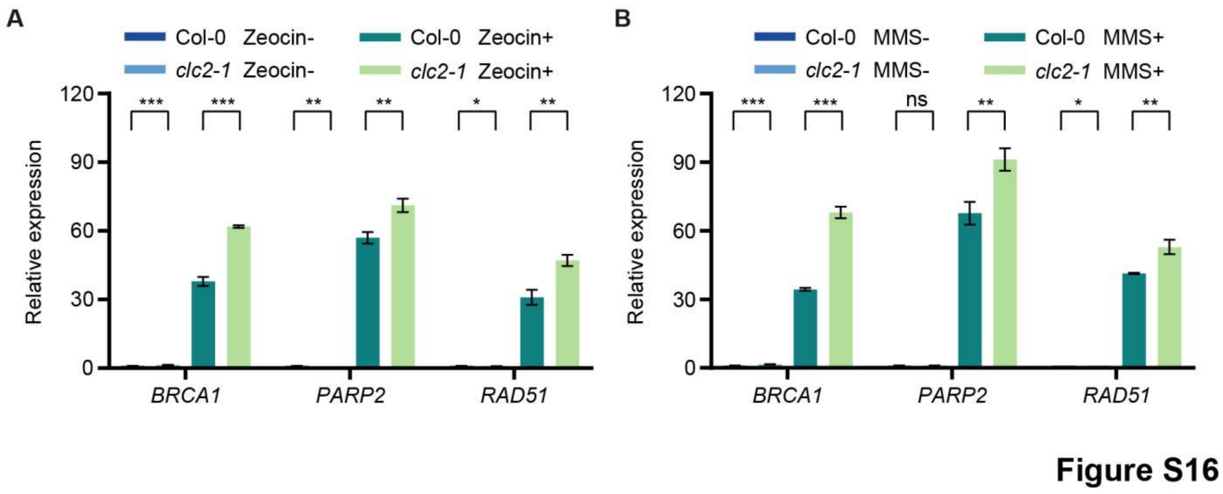

Fig. S16. Transcriptional analysis of HR related genes in wild type and *clc2-1* mutant. Seedlings were grown in 1/2MS for 7 days before treated with 10 mg/L Zeocin (A) and 50 mg/L MMS (B) for 12 h. Total RNA was extracted for qRT-PCR examination. The presented data are mean±SD from three technical replicates. Student's *t*-test. *p<0.05, **p<0.01, ***p<0.001, n.s., no significance.

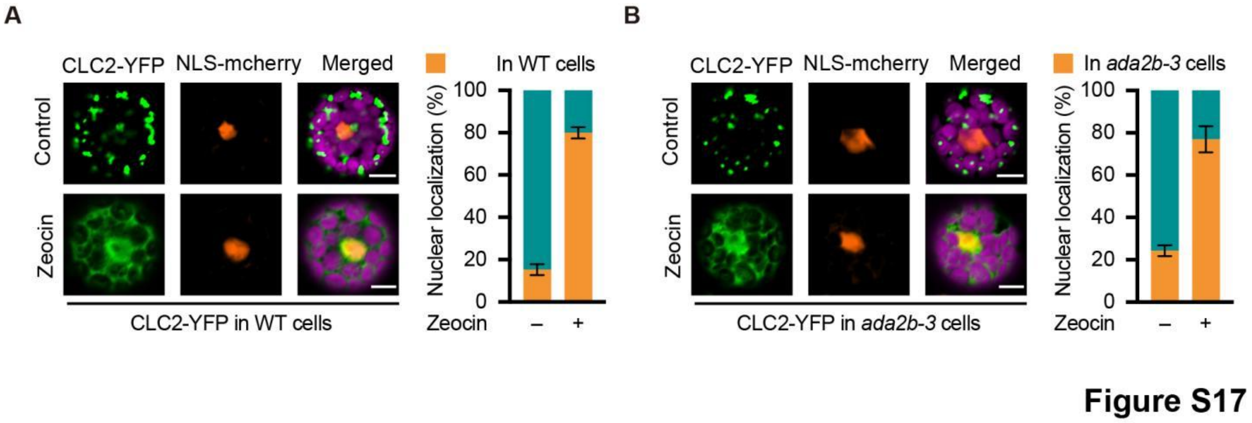

Fig. S17. Localization of CLC2-YFP in wild type and *ada2b-3* protoplasts. (**A**) Subcellular localization of CLC2-YFP in WT cells with and without DNA damage. (**B**) Subcellular localization of CLC2-YFP in *ada2b-3* cells with and without DNA damage. Representative images are shown; bars=10 μm. Percentages (mean±SD; n=100) of cells with and without nuclear localization are from three independent experiments.

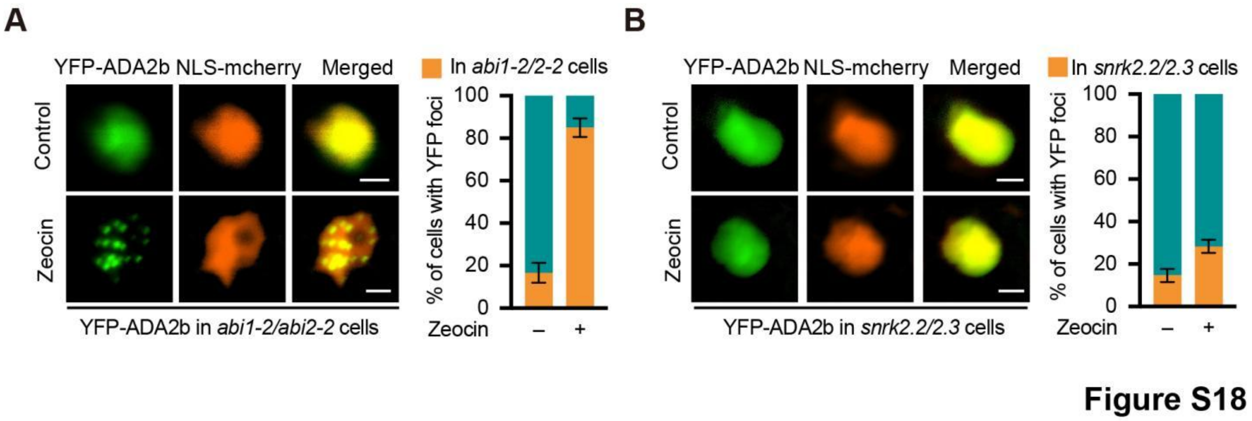

Fig. S18. Localization of YFP-ADA2b in mutants of ABA signaling pathway. (A) Subcellular localization of YFP-ADA2b in *abi1-2/abi2-2* cells with and without DNA damage. **(B)** Subcellular localization of YFP-ADA2b in *snrk2.2/snrk2.3* cells with and without DNA damage. Representative images are shown; bars=10 μm. Percentages (mean±SD; n=100) of cells with and without nuclear localization are from three independent experiments.

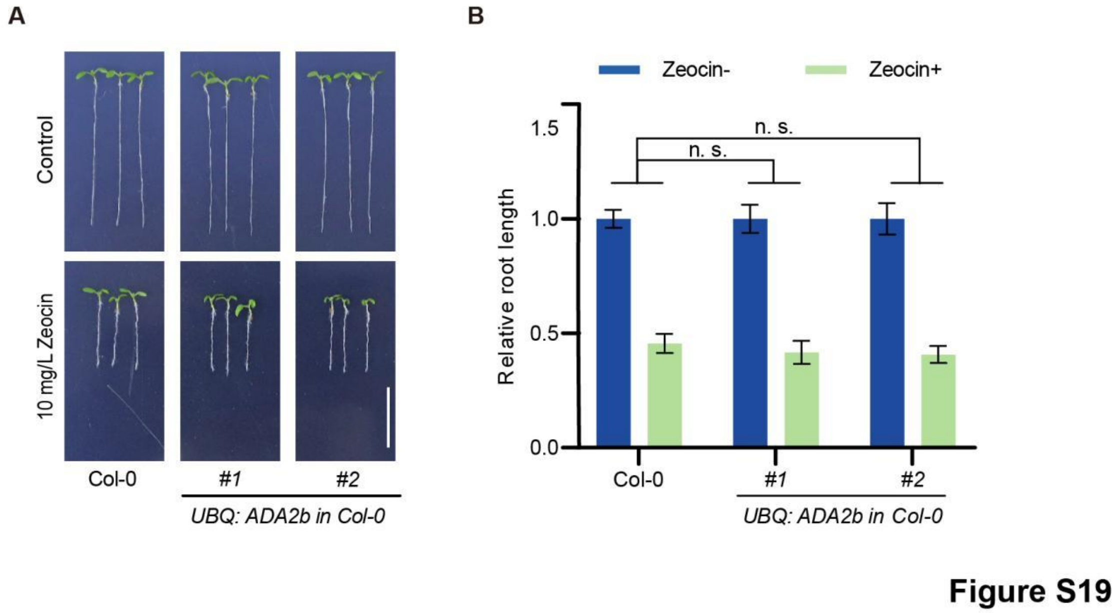

Fig. S19. Phenotypes of *YFP-ADA2b* overexpression lines in wild type seedlings under normal and DNA damage conditions. Seeds were sown on 1/2 MS medium supplemented with 10 mg/L Zeocin and grown vertically for seven days before measurements. **(A)** Representative results from three independent biological experiments are shown. Scale bar=1 cm. **(B)** Root lengths were quantified using ImageJ software, with data presented as the mean±SD (n=15). Significant differences between genotypes were determined using two-way ANOVA followed by Dunnett's multiple comparisons test. ***p<0.001.

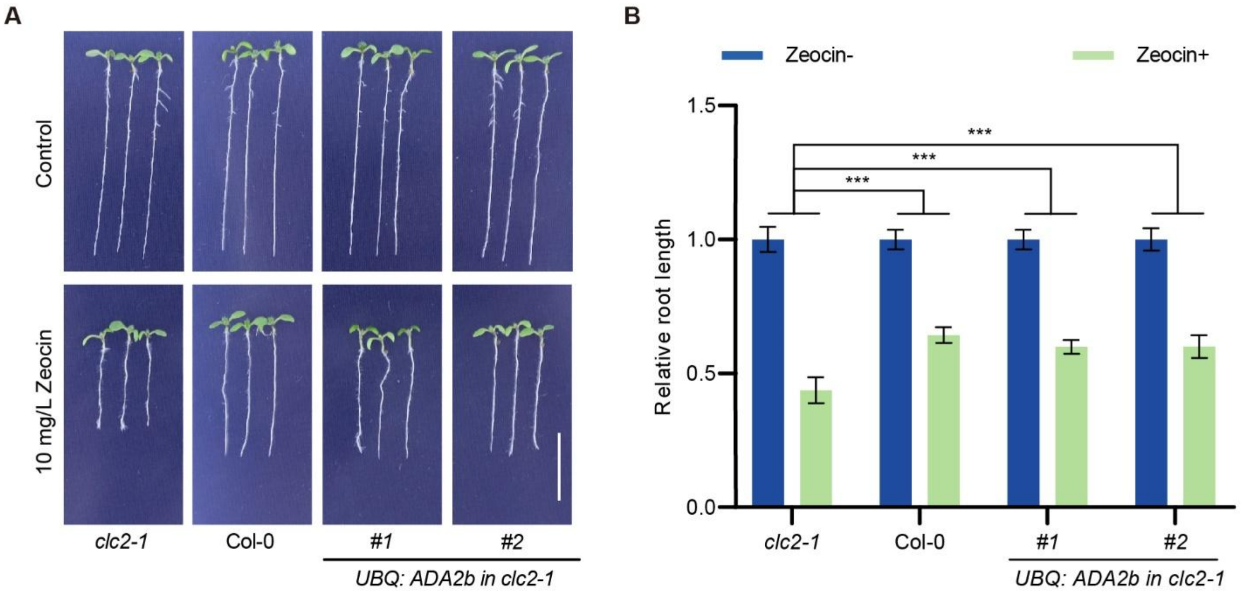

Figure S20

Fig. S20. Phenotypes of YFP-ADA2b overexpression lines in *clc2-1* seedlings under normal and DNA damage conditions. The seeds were sown on in 1/2 MS medium with 10 μg/mL Zeocin and grown vertically for seven days before recording. Three biologically independent experiments were conducted and the representative results are shown in (A); bar=1 cm. ImageJ software are used to calculate the root length and data presented as the mean±SD (n=15) are shown in (B). Significant differences between genotypes were assessed using two-way ANOVA followed by Dunnett's multiple comparisons test. ***p<0.001.

Table S1. Primers used in this study.

Supplemental Data Set 1. Primers used in this study.	
**Name**	**Sequence information 5'-3'**
UBQ:YFP-SnRK2.2-1300221-F	GCATGGACGAGCTGTACAAGGGATCCATGGATCCGGCGACTAATTCACC
UBQ:YFP-SnRK2.2-1300221-R	GACGCGTCCTAGGCTACGTAGGATCCTCAGAGAGCATAAACTATCTCTCCACTACTG
UBQ:YFP-SnRK2.3-1300221-F	GCATGGACGAGCTGTACAAGGGATCCATGGATCGAGCTCCGGTGAC
UBQ:YFP-SnRK2.3-1300221-R	GACGCGTCCTAGGCTACGTAGGATCCGAGAGCGTAAACTATCTCTCCGCT
UBQ:CLC2-YFP-1300221-F	CTGATTAACAGAAGCTTACTGTCGACATGTCTGCCTTTGAAGACGATTCCT
UBQ:CLC2-YFP-1300221-R	TCCTCGCCCTTGCTCACCATGTCGACAGCAGCAGTAACTGCCTCAGT
UBQ:CLC2A-YFP-1300221-F	GATCCTAAGAAGCCAGCAGTCGCAGTGATTCAAGGTCCA
UBQ:CLC2A-YFP-1300221-R	TGGCTTCTTAGGATCTTGTTGCTCTTTTTTTCCTCTCC
UBQ:CLC2D-YFP-1300221-F	GATCCTAAGAAGCCAGACGTCGACGTGATTCAAGGTCCA
UBQ:CLC2D-YFP-1300221-R	TGGCTTCTTAGGATCTTGTTGCTCTTTTTTTCCTCTCC
AD-SnRK2.2-F	CCATGGAGGCCAGTGAATTCATGGATCCGGCGACTAATTCAC
AD-SnRK2.2-R	GCTCGAGCTCGATGGATCCGAGAGCATAAACTATCTCTCCACTACTGTCA
AD-SnRK2.3-F	CCATGGAGGCCAGTGAATTCATGGATCGAGCTCCGGTGAC
AD-SnRK2.3-R	GCTCGAGCTCGATGGATCCGAGAGCGTAAACTATCTCTCCGC
BD-CLC2-F	TGCATATGGCCATGGAGGCCGAATTCATGTCTGCCTTTGAAGACGATTCCT
BD-CLC2-R	TGCGGCCGCTGCAGGTCGACGGATCCTTAAGCAGCAGTAACTGCCTCAGT
YFP-SnRK2.2-F	AGAGATCCCCCGGGCTGCAGGAATTCATGGATCCGGCGACTAATTCACC
YFP-SnRK2.2-R	GTATCGATAAGCTTGATATCGAATTCGAGAGCATAAACTATCTCTCCACTACTG
YFP-SnRK2.3-F	AGAGATCCCCCGGGCTGCAGGAATTCATGGATCGAGCTCCGGTGAC
YFP-SnRK2.3-R	GTATCGATAAGCTTGATATCGAATTCGAGAGCGTAAACTATCTCTCCGCT
CLC2-YFP-F	AGATCTCGAGCTCAAGCTTCGAATTCATGTCTGCCTTTGAAGACGATTCCT
CLC2-YFP-R	CCGCGGTACCGTCGACTGCAGAATTCAGCAGCAGTAACTGCCTCAGT
CLC2-CFP-F	TTTACAATTACAGTCGAGGGGGATCCATGTCTGCCTTTGAAGACGATTCCT
CLC2-CFP-R	CCCTTGCTCACCATGGCCGCGGATCCAGCAGCAGTAACTGCCTCAGT
pCDF-SnRK2.2-FLAG-F	AACTTTAATAAGGAGATATACCATGGGCATGGATCCGGCGACTAATTCAC
pCDF-SnRK2.2-FLAG-R	TCGTCATCCTTGTAATCCATAAGCTTGAGAGCATAAACTATCTCTCCACTACTG
pCDF-SnRK2.3-FLAG-F	AACTTTAATAAGGAGATATACCATGGGCATGGATCGAGCTCCGGTGAC
pCDF-SnRK2.3-FLAG-R	TCGTCATCCTTGTAATCCATAAGCTTGAGAGCGTAAACTATCTCTCCGCT
GST-CLC2-F	GACCATCCTCCAAAATCGGATGGATCCATGTCTGCCTTTGAAGACGATTCC
GST-CLC2-R	GAGTCGACCCGGGAATTCCGGGGATCCTTAAGCAGCAGTAACTGCCTCAGTG
pCDF-CLC2/CLC2A-FLAG-F	CGAGCTCGGCGCGCCTGCAGGTCGACATGTCTGCCTTTGAAGACGATTCCT
pCDF-CLC2/CLC2A-FLAG-R	TCCTTGTAATCCATAAGCTTGTCGACAGCAGCAGTAACTGCCTCAGT
CLC2-MYC-F	TTTACAATTACAGTCGAGGGGGATCCATGTCTGCCTTTGAAGACGATTCCT
CLC2-MYC-R	GCTTTGAATCGATACCGTCGGGATCCAGCAGCAGTAACTGCCTCAGT
CLC2A-YFP-F	GATCCTAAGAAGCCAGCAGTCGCAGTGATTCAAGGTCCA
CLC2A-YFP-R	TGGCTTCTTAGGATCTTGTTGCTCTTTTTTTCCTCTCC
CLC2D-YFP-F	GATCCTAAGAAGCCAGACGTCGACGTGATTCAAGGTCCA
CLC2D-YFP-R	TGGCTTCTTAGGATCTTGTTGCTCTTTTTTTCCTCTCC
CLC2 (T195A)-YFP-F	GATCCTAAGAAGCCAGCAGTCTCTGTGATTCAAGGTCCA

CLC2 (T195A)-YFP-R	TGGCTTCTTAGGATCTTGTTGCTCTTTTTTTCCTCTCC
CLC2 (S197A)-YFP-F	GATCCTAAGAAGCCAACAGTCGCAGTGATTCAAGGTCCA
CLC2 (S197A)-YFP-R	TGGCTTCTTAGGATCTTGTTGCTCTTTTTTTCCTCTCC
nYFP-ADA2b-F	CAACATCGAGGACaGAATTCGTCGACatATGGGTCGCTCTCGAGGGAA
nYFP-ADA2b-R	GGGGAATTACTAGTCCCGGGGTCGACAAGTTGAGCAATACCCTTCTTCACAAG
CLC2-cYFP-F	GTCGAGGGGGATCCCTCGAGAAGCTTATGTCTGCCTTTGAAGACGATTCCT
CLC2-cYFP-R	CTGCACGCTGCCgtCCATGGAAGCTTgAGCAGCAGTAACTGCCTCAGT
ACTIN2-realtime-F	GGTAACATTGTGCTCAGTGGTG
ACTIN2-realtime-R	CTCGGCCTTGGAGATCCACATC
PARP2-realtime-F	ATGGCGTTCTGCTCCTCTGC
PARP2-realtime-R	GGTGCTGTTTTCCCCACACC
BRCA1-realtime-F	CCATGTATTTTGCAATGCGTG
BRCA1-realtime-R	TGTGGAGCACCTCGAATCTCT
RAD51-realtime-F	CGAGGAAGGATCTCTTGCAG
RAD51-realtime-R	GCACTAGTGAACCCCAGAGG
KU70-realtime-R	CGAGGACGACGTTGCAGAGAGC
KU70-realtime-F	GCTGCCAGGAATAGCCGGACG
KU80-realtime-R	GATGATGAAGACAATCGCATGATTA
KU80-realtime-F	TTAGCTCTCGAGCATTGACTC
XRCC4-realtime-F	GCGCAAGGTGAAAAACTCTGT
XRCC4-realtime-R	TTGAGGCTTCTTCCTCGCTC
RD29A-realtime-F	TTCCACCAGGGACAAAGGTG
RD29A-realtime-R	GGTGCATCGTGTCCGTAAGA
RD29B-realtime-F	GAAACCAAAGATGAGTCGACAC
RD29B-realtime-R	TTTTTCGTAAACCGGAGTCAAC
PAO-realtime-F	TCACTCCAACCCAGGCAGAC
PAO-realtime-R	GATAAACCAGCAAGAACCAGTCG
NYE1-realtime-F	GCAAGGATGGGCAAATAGG
NYE1-realtime-R	CACCGCTTATGTGACAATGAAC
NYE2-realtime-F	GACGAAGTAGTGGGCGAGTG
NYE2-realtime-R	CGATGAGATTCAAGAAGAAGTGG
SAG29-realtime-F	CTGTTTTCGCTGCCCCTCT
SAG29-realtime-R	ACAGCCCTAGTACGAATCCCAC
CLC2-realtime-F	TCGACGATGTTTTTGCAGCG
CLC2-realtime-R	GGCAAGATAGGACCGTCGTG
SnRK2.2-realtime-F	ATGTGATTTTGGTTATTCCAAGGT
SnRK2.2-realtime-R	TGCAATGTATGCAGGAGTACCA
SnRK2.3-realtime-F	CGCTCCAGAGGTACTGCTTC
SnRK2.3-realtime-R	CGGATACGCTCCAACCAACA
clc2-1 (Salk_016049)-LP	GCCATAGCACGAAATCAGATC
clc2-1 (Salk_016049)-RP	GCTCAATGATTGTGCCAATTC
snrk2.2 (GABI-Kat 807G04)-RP	CCTAATATTGTTAGGTTTAAA
snrk2.2 (GABI-Kat 807G04)-LP	GCAAGACCATACATCTGCAAG
snrk2.3 (Salk_096546)-LP	GGTTTTGAGTGTTCTGCTTTTG

snrk2.3 (Salk_096546)-RP	ACATCTGCAATCTGGTAACCG
snrk2.6 (Salk_008068)-LP	CATATCTTTAGACGAGGGGCC
snrk2.6 (Salk_008068)-RP	GTGAGTGGTCCAATGGATTTG
abi1-2 (Salk_072009)-LP	TGAATATAGGAAGTCTGAAGCAAGTG
abi1-2 (Salk_072009)-RP	CGAAACAGCATCTTCCATCTC
abi2-2 (Salk_015166C)-LP	AAACTGTTGGGTCTACCTCGG
abi2-2 (Salk_015166C)-RP	ACCATCCCATATTCTGGTTGG
ada2b-3 (Salk_019407)-RP	CTCAGTAGTGCCAGCCTTTTG
ada2b-3 (Salk_019407)-LP	AAACCAAACGCACAGTTTGAG
LBb1.3	ATTTTGCCGATTTCGGAAC
T-DNA primer for snrk2.2	ATAATAACGCTGCGGACATCTACATTTT
